# Untargeted and Targeted Metabolomics and Tryptophan Decarboxylase In Vivo Characterization Provide Novel Insight on the Development of Kiwifruits (*Actinidia deliciosa*)

**DOI:** 10.3390/ijms20040897

**Published:** 2019-02-19

**Authors:** Mauro Commisso, Stefano Negri, Martino Bianconi, Sofia Gambini, Sara Avesani, Stefania Ceoldo, Linda Avesani, Flavia Guzzo

**Affiliations:** 1 Department of Biotechnology, University of Verona, Strada Le Grazie 15, 37134 Verona, Italy; stefano.negri@univr.it (S.N.); martinobianconi@dembiotech.it (M.B.); sofia.gambini@studenti.univr.it (S.G.); saraavesanivr@gmail.com (S.A.); stefania.ceoldo@univr.it (S.C.); linda.avesani@univr.it (L.A.); flavia.guzzo@univr.it (F.G.); 2 Demethra Biotech, Strada dell’Innovazione 1, Camisano Vicentino, 36043 Vicenza, Italy

**Keywords:** *Actinidia deliciosa*, untargeted metabolomics, abscisic acid, tryptamine, serotonin, tryptophan decarboxylase

## Abstract

Kiwifruit (*Actinidia deliciosa* cv. Hayward) is a commercially important crop with highly nutritional green fleshy fruits. The post-harvest maturation of the fruits is well characterized, but little is known about the metabolic changes that occur during fruit development. Here we used untargeted metabolomics to characterize the non-volatile metabolite profile of kiwifruits collected at different time points after anthesis, revealing profound metabolic changes before the onset of ripening including the depletion of many classes of phenolic compounds. In contrast, the phytohormone abscisic acid accumulated during development and ripening, along with two indolamines (serotonin and its precursor tryptamine), and these were monitored in greater detail by targeted metabolomics. The role of indolamines in kiwifruit development is completely unknown, so we also characterized the identity of genes encoding tryptophan decarboxylase in *A. deliciosa* and its close relative *A. chinensis* to provide insight into the corresponding biological processes. Our results indicate that abscisic acid and indolamines fulfill unrecognized functions in the development and ripening of kiwifruits.

## 1. Introduction

The common green kiwifruit (*Actinidia deliciosa*) is a climacteric berry cultivated mainly in China, Italy, New Zealand, Iran, Chile, and Greece, with annual production exceeding four million tons [1]. The widely-consumed fresh fruits are rich in vitamins C, E, and K, as well as calcium, magnesium, and potassium [2]. Furthermore, supplements based on kiwifruits are used to promote gut health [3,4,5,6,7]. Fruits are perceived as rich in fiber, but the fiber content is only 3% of fresh weight (FW), so kiwifruits cannot be classed as a high-fiber food [2]. Kiwifruits are also thought to provide antioxidant and anti-inflammatory compounds [8] and metabolites that improve sleep quality [9,10].

For commercial purposes, kiwifruits are usually harvested as unripe fruit. Ripening occurs during post-harvest storage when fruit softening and soluble sugar accumulation make the fruit edible [11]. The ripening process is also accompanied by the accumulation of volatiles [12]. The accumulation of sugars, organic acids, and volatiles has been investigated during kiwifruit development and post-harvest ripening as hallmarks of the ripening process and in terms of their impact on flavor. Sucrose and the trisaccharide planteose (galactosucrose) are the major sugars translocated to the fruits during development [13]. Subsequent ripening involves a complex and highly dynamic process of simultaneous starch accumulation and degradation that culminates in the conversion of starch to sucrose [14]. Glucose, fructose and sucrose accumulate in the fully-ripe fruit [15]. Common organic acids such as citric and malic acid also accumulate during fruit development and ripening, where they control the osmotic balance [16], but free quinic acid is also abundant in ripe kiwifruits, although rare in other species [15]. Many volatiles also accumulate during ripening, including aldehydes, alcohols, esters, ketones, norisoprenoids monoterpenes, and other hydrocarbons [12].

The accumulation of these primary and secondary metabolites during ripening has a strong impact on flavor perception. Dry matter, volatiles, and the correct balance between sugars and acids are considered necessary for preferred kiwifruit flavor attributes [17,18]. The post-harvest change in firmness is also important for taste perception [19]. The control of kiwifruit ripening has also been investigated. As in other climacteric fruits, kiwifruit ripening involves a climacteric phase characterized by an increase in respiration, CO_2_ consumption and ethylene accumulation [11,20]. The climacteric respiration burst occurs after the completion of starch degradation and in turn controls the accumulation of volatiles and late (from fully-ripe to over-ripe) fruit softening [20]. Kiwifruits undergo natural ripening during the cold season, so chilling can also trigger the ripening process, and a positive interaction between ethylene and chilling has been observed [21]. Abscisic acid (ABA) is likely to play a role in kiwifruit ripening because the fruits produce ethylene in response to exogenous ABA [22].

The development and ripening of fleshy fruits also tends to involve the profound modification of the profile of non-volatile secondary metabolites. In many plant species, these metabolites play a crucial role in defense against pests/herbivores or the attraction of animals for seed dispersion [23]. If the accumulation of toxic, defense-related metabolites is desirable in the immature fleshy fruit, they should be at least partially converted to less toxic compounds in fully-ripe fleshy fruit to favor consumption by seed dispersers. For example, the profile of phenolic compounds and alkaloids during development has been extensively investigated in model species such as tomato [24]. The transition from green fruits to the breaker and red stages is characterized by the loss of caffeoyl quinic acid, lycoperoside and α-tomatine (the latter being toxic for many animals) and the accumulation of esculeoside A, quercetins and phloretins [25,26]. In kiwifruit, non-volatile secondary metabolites have been studied mainly to determine the health-promoting properties of mature fruits and fruit juices [27,28], but their role in development is unclear.

Here we carried out untargeted and targeted metabolomics analysis to characterize the dynamic metabolic profile of kiwifruits during development and to gain insight into the role of specific metabolites. The untargeted metabolomics approach revealed the accumulation of certain non-volatile secondary metabolites during fruit development, including polyphenols, indolamines and the phytohormone ABA, each showing specific developmental profiles. We then used targeted metabolomics with deuterated authentic standards to measure the levels of these key compounds more precisely, revealing the unique accumulation profiles of tryptamine and serotonin, whose role in fruit development is completely unknown. Tryptamine is produced by the enzyme tryptophan decarboxylase (TDC), and in some species it is channeled directly into the indolalkaloid pathway whereas in others it is the precursor of serotonin [29,30]. We therefore characterized the expression and activity of TDC genes in the related species *A. deliciosa* and *A. chinensis* as a basis for future investigations to determine the biological roles of tryptamine and serotonin during kiwifruit development.

## 2. Results

### 2.1. Sampling Plan

**Figure 1:**
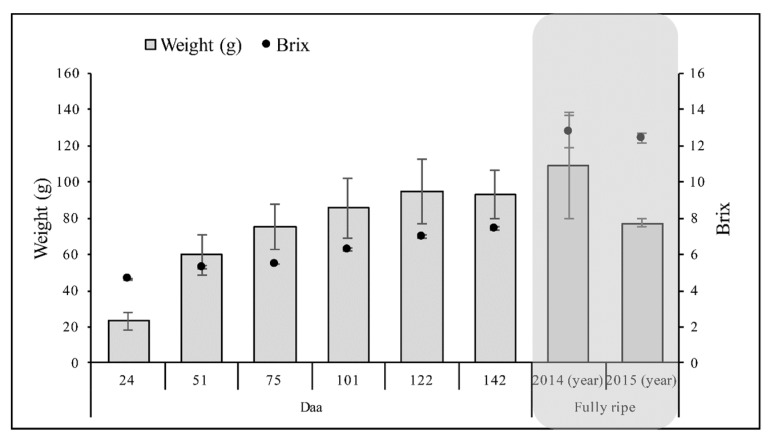
Characterization of developing and fully-ripe *Actinidia deliciosa* cv Hayward kiwifruits. Columns and circles indicate the fruit weight (grams) and the degrees Brix (percentage soluble sugar). The gray box shows the fully-ripe fruits. Data are means and standard deviations (*n* = 3), daa = days after anthesis.

*Actinidia deliciosa* cv. Hayward fruits were collected during 2016 from five different plants widely distributed along two rows in an orchard near Verona (Italy). The fruits were collected 24, 51, 75, 101, 122, and 142 days after anthesis (daa) and three sample pools of 20 fruits were prepared for each time point. The last time point corresponds to the day kiwifruits are commercially harvested, and was established according to the soluble sugar content (degrees Brix), as specified by the farmers. Fully ripe kiwifruits were also included in the analysis, and three sample pools were created, each containing 50 fruits. After transfer to the laboratory, the fruits were weighed and the soluble sugar content was determined (Figure 1).

### 2.2. Untargeted LC-MS Analysis of Kiwifruits during Development

**Figure 2:**
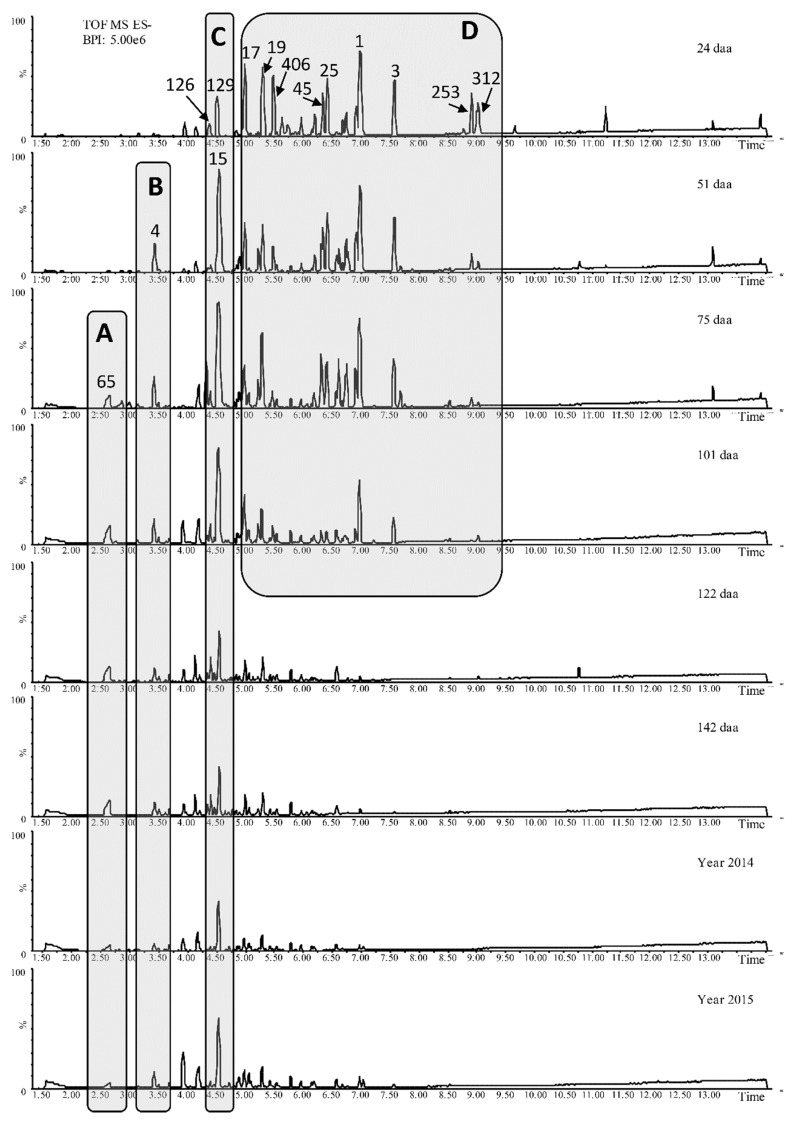
Untargeted metabolomics (UPLC-qTOF) negative base peak chromatograms of kiwifruit methanol extracts (daa = days after anthesis). Younger kiwifruits are listed from the top and the numbers above each peak show the corresponding molecular ID (Supplementary File 1). Box A highlights the homogluthatione profile (first detected 75 daa and the content remained stable throughout the rest of development, resulting in a shorter peak in fully-ripe fruits). Box B highlights the similar trend for caffeoyl sucrose, although this metabolite appeared earlier (51 daa). Box C highlights the profiles for benzoyl glucuronide and coumaroyl quinic acid (ID 126, 129), which were already present in the earliest sample (24 daa), and caffeic acid hexose (ID 15), which arose at 51 daa and remained stable until 101 daa before decreasing. Box D highlights the profiles of several flavan-3-ols (ID 17, 19, 406), flavonols (ID 45, 25, 1, 3) and terpenes (ID 253, 312) that were already present in the first sample and then declined in abundance, almost disappearing at 122 daa.

The samples were powdered in liquid nitrogen and methanol extracts were analyzed by untargeted metabolomics (UPLC-qTOF). The base peak chromatograms clearly showed the profound changes in the secondary metabolome during kiwifruit development (Figure 2).

**Figure 3:**
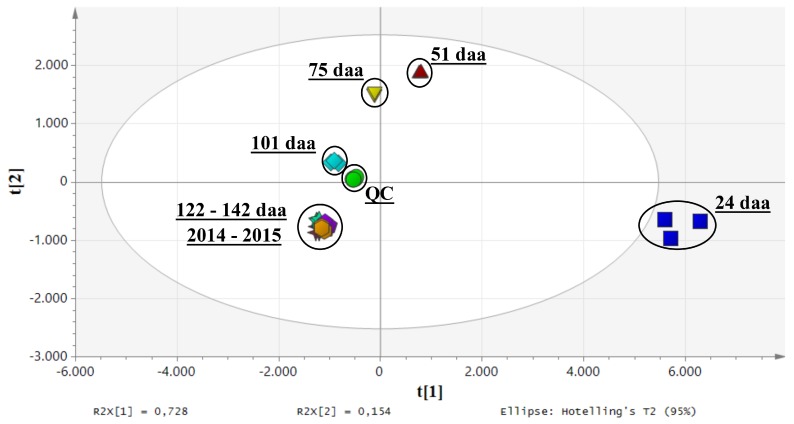
PCA score scatter plot of kiwifruit samples collected at different time points after anthesis. The clustering depends on secondary metabolites. QC stands for quality control.

The high-resolution untargeted metabolomics analysis produced a data matrix comprising 521 putative compounds, 81 of which were tentatively annotated (Supplementary File 1). The dataset was submitted to PCA analysis and the first and second principal components, explaining the 88.2% of the total variance, showed sample clustering according to the developmental stage (Figure 3).

**Figure 4:**
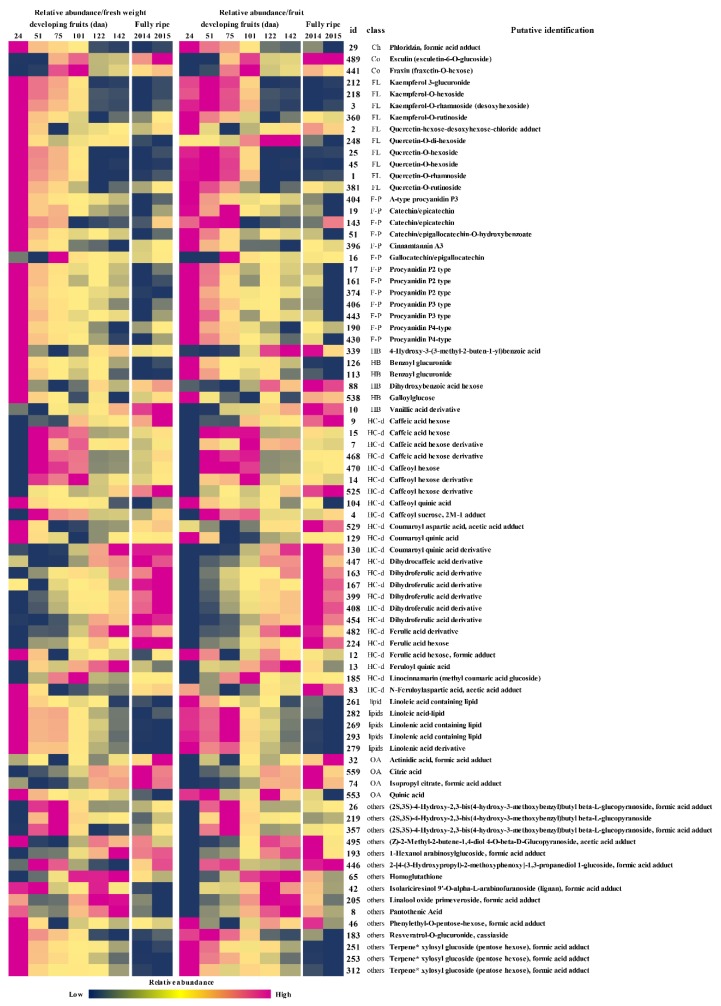
Heat map showing the average relative abundance as a proportion of fresh weight (calculated from the dataset in Supplementary File 1) and the average relative abundance per fruit (obtained by normalizing the relative abundance as a proportion of fresh weight by the average fruit weight) of all the metabolites tentatively identified during fruit development and in fully-ripe fruits, the latter from two different growing seasons. The metabolite identification numbers (ID) match those in the dataset. The color code ranges from blue (lowest relative abundance to magenta (highest relative abundance). Abbreviations: Co = coumarins; Ch = chalcones; FL = flavonoids; F-p = flavan-3-ols and procyanidins; HB = hydroxybenzoic acid derivatives; HC-d = hydroxycinnamic acid derivatives; OA = other organic acids. * The *m*/*z* and fragmentation pattern of the terpene moiety was compatible with linalool, nerol or geraniol.

**Figure 5:**
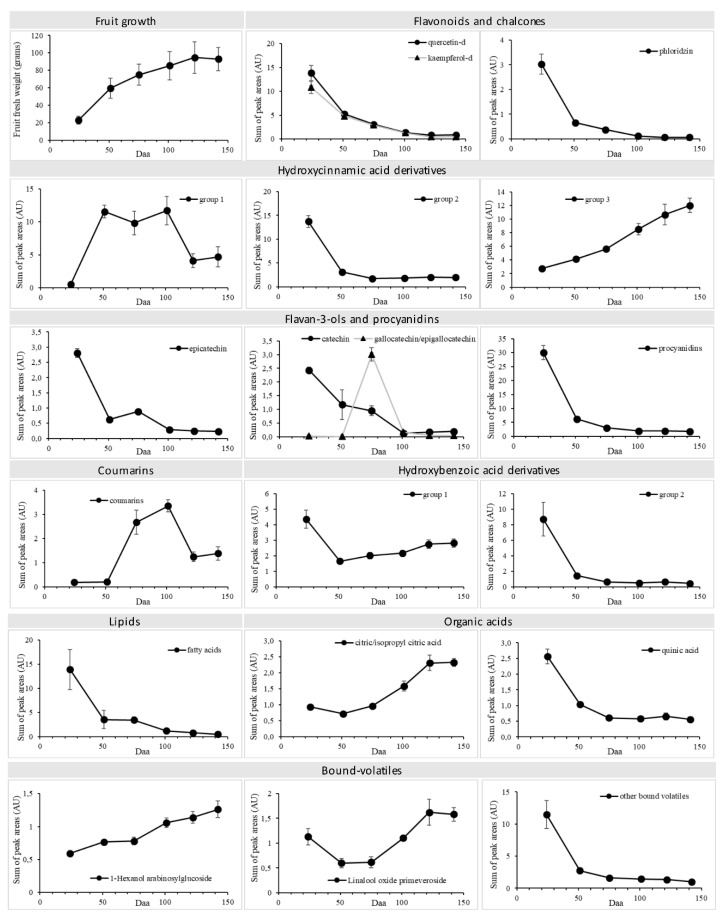
Fruit growth curve and relative abundance (means ± standard deviation) of selected metabolites/groups of metabolites during fruit development. The original dataset (Supplementary File 1) was autoscaled (UV). The IDs are the same as shown in Figure 4. Hydroxycinnamic acids, group 1: ID 9 caffeic acid hexose, ID 15 caffeic acid hexose, ID 7 caffeic acid hexose derivative, ID 468 caffeic acid hexose derivative, ID 14 caffeoyl hexose derivative, ID 525 caffeoyl hexose derivative, ID 470 caffeoyl hexose, ID 4 caffeoyl sucrose, ID 185 linocinnamarin (methyl coumaric acid glucoside). Hydroxycinnamic acids, group 2: ID 104 caffeoyl quinic acid, ID 529 coumaroyl aspartic acid, ID 129 coumaroyl quinic acid, ID 83 *N*-feruloylaspartic acid, ID 12 ferulic acid hexose formic adduct. Hydroxycinnamic acids group 3: ID 130 coumaroyl quinic acid derivative, ID 482 ferulic acid derivative, ID 224 ferulic acid hexose, ID 13 feruloyl quinic acid, ID 163 dihydroferulic acid derivative, ID 167 dihydroferulic acid derivative, ID 399 dihydroferulic acid derivative, ID 408 dihydroferulic acid derivative, ID 454 dihydroferulic acid derivative, ID 447 dihydrocaffeic acid derivative. Hydroxybenzoic acids group 1: ID 10 vanillic acid derivative, ID 88 dihydroxybenzoic acid hexose, ID 339 4 hydroxy-3-(3-methyl-2-buten-1-yl) benzoic acid. Hydroxybenzoic acids group 2: ID 113 benzoyl glucuronide; ID 126 benzoyl glucuronide, ID 538 galloylglucose.

Among the detected metabolites, the most relevant groups included hydroxycinnamic acid derivatives (~39% of the annotated metabolites), flavan-3-ols and procyanidins (17%), flavonoids (15%), and hydroxybenzoic acid derivatives (10%). The other classes of metabolites included coumarins, lipids, other organic acids, and putative aroma precursors. A heat map of the annotated metabolites at different stages of fruit development and in fully-ripe fruits over two different growing seasons is shown in Figure 4, and the relative abundance of selected metabolites/groups of metabolites during fruit development is summarized in Figure 5.

The relative abundance of most of the metabolites followed a developmental trend of accumulation or depletion. The fully-ripe fruits represented two growing seasons, and were used only to confirm the persistence of the metabolites in the ready-to-eat ripe fruits, irrespective of the accumulation/depletion dynamics. The most dynamic metabolite profiles were generally observed during the early phase of very rapid growth, from the first to the second sampling points, when the fruit weight increased to ~50 g. Metabolites with an early accumulation phase followed by a late depletion phase (such as the caffeic acid glycosides and coumarins) were depleted more rapidly from the fourth sampling point, when fruit growth was almost complete (Figure 4 and Figure 5).

Coumarins: The two coumarins we detected (esculin and fraxins) showed an accumulation-depletion profile and persisted in the fully-ripe fruits.

Flavonoids: The flavonoids we detected included quercetin and kaempferol based-flavonoids, all of which were depleted during development, albeit at different rates. Quercetin-*O*-rhamnoside was the most abundant flavonoid at the beginning of fruit development and the most persistent in the fully ripe fruits. Kaempferol-*O*-rhamnoside and the quercetin and kaempferol rutinosides were also persistent. Only quercetin-*O*-dihexoside showed a slight increase in abundance per fruit during development (Figure 4 and Figure 5).

Hydroxycinnamic acid derivatives: This class of compounds mainly comprised glycosides, esters, and amides of caffeic, coumaric, and ferulic acids, as well as some derivatives of dihydrocaffeic and dihydroferulic acid, and it showed a more complex accumulation-depletion profile: one group (the quinic esters of caffeic and coumaric acid, the aspartic amides of coumaric and ferulic acid, and one ferulic acid hexose) declined during fruit development, especially during early growth. Another group, including all the glucosides of caffeic acid, showed a rapid increase followed by depletion. The remaining compounds in this class (coumaric, ferulic, dihydroferulic, and dihydrocaffeic acid derivatives) increased linearly during development and persisted in the fully-ripe fruit.

Hydroxybenzoic acid derivatives: These metabolites showed a sharp decline during early growth followed by a slower decline or in some cases a slight increase.

Flavan-3-ols and procyanidins: These metabolites declined during fruit development. In particular, gallocatechin/epigallocatechin was abundant (although only from the third sampling stage) and then decreased sharply.

Lipids: All of the detected lipids gradually decreased in abundance during fruit development.

Other organic acids: Citric and isopropyl citric acid became more abundant during development whereas the level of quinic acid declined.

Among the other metabolites, the most interesting profiles were the increasing levels of homoglutathione and the detection of putative aroma precursors, namely three terpene-pentose hexoses (with *m*/*z* values and fragmentation patterns compatible with linalool, nerol or geraniol), 1 hexanol arabinosylglucoside, linalool oxide primeveroside and phenylethyl-*O*-pentose-hexose. The terpene and phenylethyl pentose hexose declined during fruit development while the other two accumulated. The linalool oxide, 1-hexanol and phenyl ethyl glycosides were the most persistent metabolites in the fully-ripe fruits.

### 2.3. Assessment of ABA Levels in Developing Fruits

**Figure 6:**
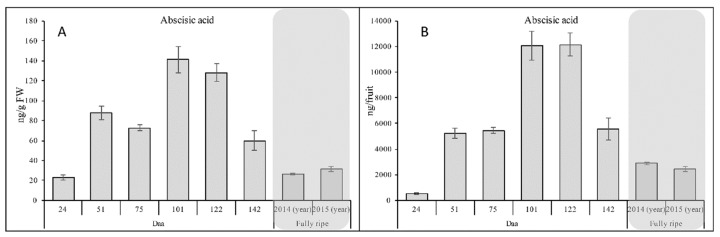
Abscisic acid (ABA) levels in kiwifruits at different developmental stages and in fully-ripe fruit. (**A**) Abundance of ABA (ng) per gram of fresh weight. (**B**) Abundance of ABA (ng) in a whole fruit with skin removed. Gray boxes highlight the values for fully-ripe fruits.

The application of high-resolution UPLC-MS in the untargeted metabolomics approach allowed the detection of ABA in the developing fruits (Figure S1 in Supplementary File 2). This phytohormone is known to be involved in fruit development and ripening [31,32] and therefore we investigated the accumulation of this important molecule in relation to kiwifruit development. Although we detected ABA in the original methanol extracts, we also applied a specific extraction protocol to allow more precise measurements [33]. The sensitive UPLC-MS method allowed us to detect ABA at the first sampling point (24 daa) at a concentration of 22.94 ng/g FW, followed by an increase at 51 daa and then a decrease at 75 daa (Figure 6A). However, when the values were normalized to the fruit weight, the difference between these two phases was eliminated (Figure 6B). Similarly, we observed an ABA peak at 101 daa followed by a slight decline at 122 daa (Figure 6A) but the difference was eliminated when the fruit weight was taken into account (Figure 6B). The levels of ABA declined after 122 daa, reaching a value higher than that observed at 24 daa. Significant ABA quantities were still observed in fully ripe fruits.

### 2.4. Assessment of Tryptamine and Serotonin Levels in Developing Fruits

**Figure 7:**
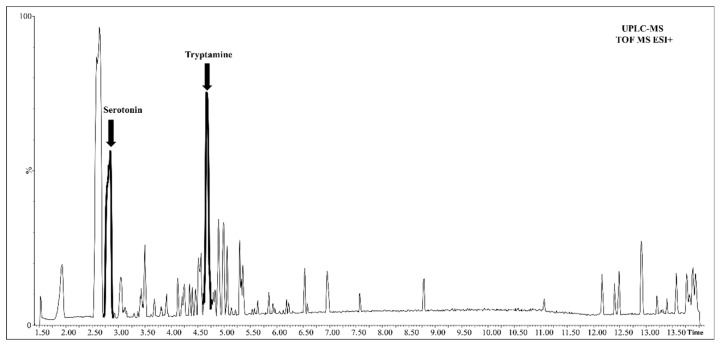
UPLC-MS chromatogram of metabolites in positive ionization mode. The arrows indicate serotonin and tryptamine, whose abundance is indicated by the high peaks.

Next we analyzed the kiwifruit samples to determine the levels of tryptamine and serotonin. The chromatographic peak corresponding to serotonin was one of the highest peaks detected in positive ionization mode and it eluted at 2.84 min, whereas its precursor tryptamine yielded an even higher peak and eluted at 4.68 min (Figure 7).

**Figure 8:**
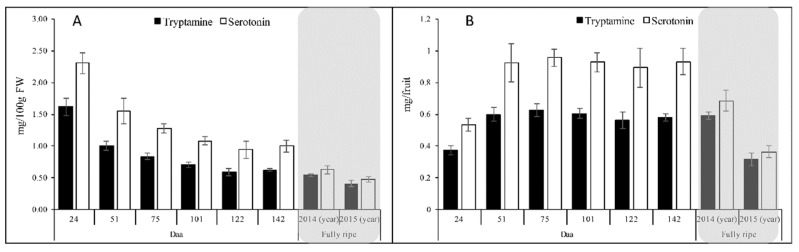
Tryptamine and serotonin levels in kiwifruits at different developmental stages and in fully-ripe fruit. (**A**) Abundance of tryptamine and serotonin (ng) per gram of fresh weight. (**B**) Abundance of tryptamine and serotonin (ng) in a whole fruit with skin removed. Gray boxes highlight the values for fully-ripe fruits.

Both metabolites were highly abundant throughout development, as confirmed by comparing serotonin and tryptamine in the fruit extracts against the corresponding authentic commercial deuterated standards. The dynamic developmental profiles of tryptamine and serotonin were similar when compared as a proportion of fruit fresh weight (Figure 8A) or in terms of the quantity present per fruit (Figure 8B).

As a proportion of fruit fresh weight, the levels of tryptamine and serotonin were very high at the earliest sampling point (24 daa) indicating that the synthesis of these compounds is activated following anthesis. The content of both metabolites then declined progressively, leveling off at 101 daa. In fully-ripe fruits, both tryptamine (0.48 ± 0.04 g/100 g FW) and serotonin (0.55 ± 0.05 g/100 g FW) levels were assessed, indicating that kiwifruits ready-to-eat accumulate these two indolamines (Figure 8A). When considered in terms of quantity per whole fresh fruit with the skin removed, the initial levels of serotonin and tryptamine increased following anthesis and then remained fairly stable from 51 days onwards (Figure 8B). Interestingly, in the fully-ripe fruits, the tryptamine and serotonin levels were influenced by the growing season: in 2015 the levels of both tryptamine and serotonin declined respect to 2014.

### 2.5. Characterization and Transient Expression of Kiwifruit TDC

#### 2.5.1. Sequence Analysis

The high levels of tryptamine in *A. deliciosa* kiwifruits suggested that the enzyme TDC was present and active, allowing the conversion of tryptophan into tryptamine. In plants which accumulate tryptamine and serotonin, the highest levels of these molecules are found in fruits, but their role in fruit development is completely unknown. The expression profile of the *TDC* gene may provide some insight into this process, so we used the *Catharanthus roseus* ortholog (CrTDC) to identify matching sequences in the Kiwifruit Genome Database, which includes genetic resources for *A. chinensis* [34]. The results are shown in Table 1.

InterProScan analysis suggested that these sequences belong to the aromatic amino acid decarboxylase (AADC) family, a group of enzymes with pyridoxal phosphate (PLP) as a co-factor. Moreover, the TargetP analysis showed high scores for ‘any other location’, suggesting that these putative enzymes are likely be cytosolic. Achn173261 was most similar to CrTDC, and was therefore selected for further characterization.

#### 2.5.2. Characterization of *A. deliciosa* TDC

**Figure 9:**
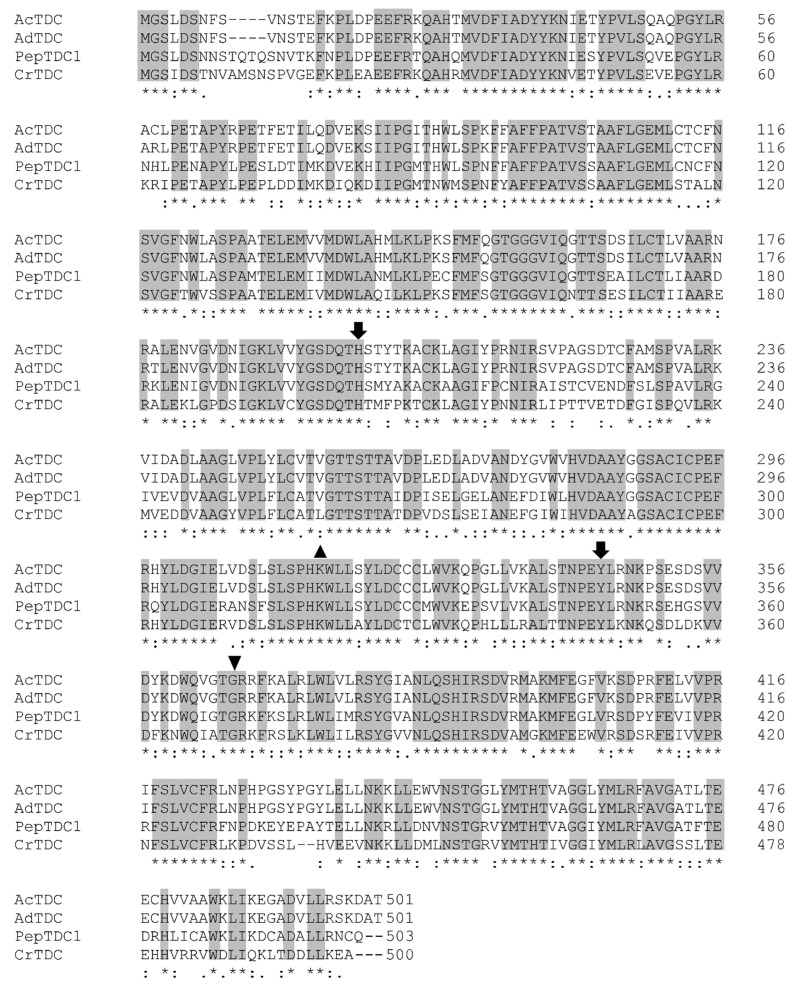
Clustal Omega alignment of Achn173261, AdTDC11, PepTDC1, and CrTDC amino acid sequences. Gray boxes indicate conserved residues. Symbols indicate the putative pyridoxal phosphate-binding site (triangle), the putative catalytic residues (arrows), and the putative substrate selectivity residue (inverted triangle).

By amplifying cDNA prepared from fully-ripe kiwifruit flesh, 20 candidate *A. deliciosa TDC* (*AdTDC*) sequences were recovered (Figure S2 in Supplementary File 2). Among the candidates, *AdTDC11* was most similar to *Achn173261* (data not shown) and a ClustalW alignment of the two deduced amino acid sequences revealed an identity of 99.2% and a similarity of 99.6%, with only four mismatched amino acids (Figure S3 in Supplementary File 2). The deduced Achn173261 and AdTDC11 amino acid sequences were also aligned with the functionally characterized PepTDC1 from pepper [35] and CrTDC [29]. This revealed many conserved regions, including a putative pyridoxal phosphate (PLP) binding site, two catalytic residues and a substrate selectivity residue (Figure 9). These enzymatic features of CrTDC were recently confirmed by X-ray diffraction [36].

#### 2.5.3. Phylogenetic Analysis

**Figure 10:**
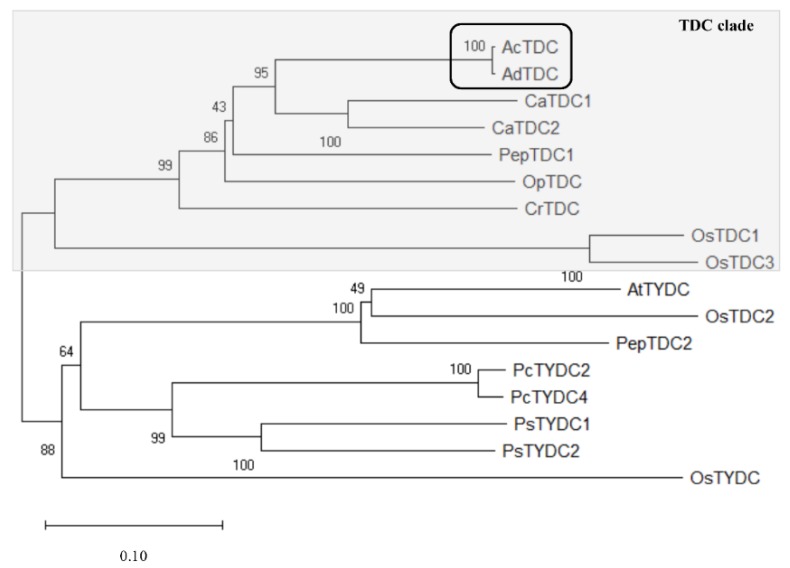
Phylogenetic tree including the amino acid sequences of *A. chinensis* and *A. deliciosa* tryptophan decarboxylases (TDCs). The tree was based on the amino acid sequences previously reported [37]. MUSCLE was used for sequence alignment and a neighbor-joining algorithm was used to construct the tree in Mega-X. Numbers next to the branches indicate the replicate percentage calculated using the bootstrap method (1000 replicates).

The amino acid sequences of some functionally characterized TDCs, together with another class of AADC, i.e., the tyrosine decarboxylases (TYDCs), were used to construct a phylogenetic tree (Figure 10). Achn173261 and AdTDC clustered in the TDC clade, indicating these two genes were highly likely to encode TDCs rather than TYDCs.

#### 2.5.4. Characterization of Recombinant *Actinidia* spp. TDCs

**Figure 11:**
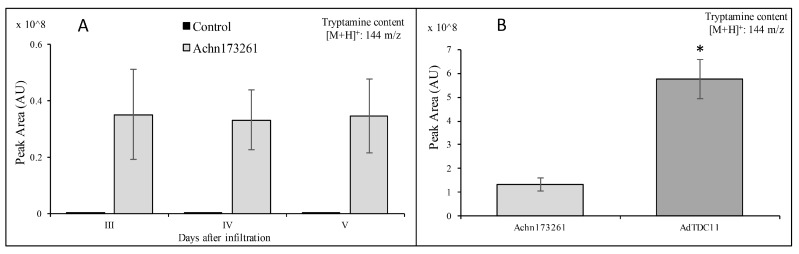
HPLC-ESI/MS analysis confirming the presence of tryptamine in infiltrated *Nicotiana benthamiana* plants. (**A**) Tryptamine content in *N. benthamiana* leaves 3, 4, and 5 days after infiltration with pK7WG2.Achn173261. (**B**) Tryptamine content in infiltrated *N. benthamiana* leaves 4 days after infiltration with either pK7WG2.Achn173261 or pK7WG2.AdTDC11 (* *p* < 0.01).

*Achn173261* and *AdTDC11* were functionally characterized by inserting the coding sequences downstream of the Cauliflower mosaic virus 35S promoter in the binary GATEWAY vector pK7WG2. The resulting vectors pK7WG2.Achn173261 and pK7WG2.AdTDC were individually introduced into *Agrobacterium tumefaciens* and the bacterial suspensions were infiltrated into three leaves each on three different *Nicotiana benthamiana* plants, which do not naturally produce tryptamine. After harvesting the three leaves on sequential days, methanol extracts were prepared and analyzed by untargeted metabolomics (HPLC-ESI/MS). The analysis of pK7WG2.Achn173261 infiltrated plants revealed the accumulation of tryptamine 3 days after infiltration and the level remained stable for the next 2 days (Figure 11A). The analysis of leaves collected 4 days after infiltration also confirmed the production of tryptamine in plants infiltrated with pK7WG2.AdTDC11 (Figure 11B). Interestingly, the levels of tryptamine in those plants were at least four-fold higher than in plants transiently expressing *Achn173261* (Figure 11B).

#### 2.5.5. *AdTDC* Expression at the Onset of Fruit Development

**Figure 12:**
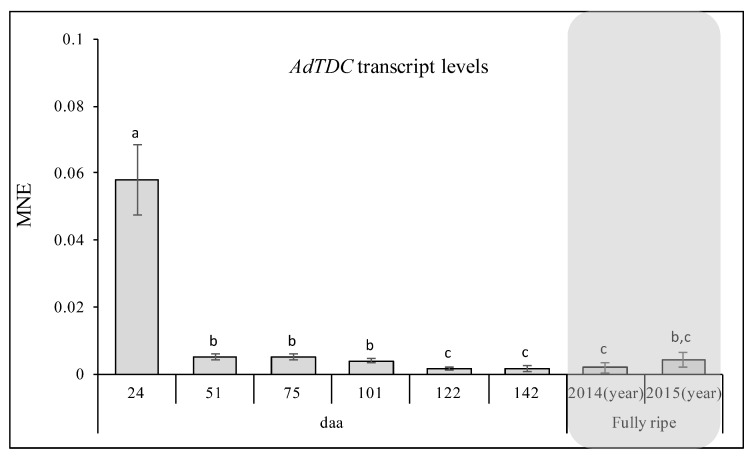
Expression level of the *AdTDC* gene determined by quantitative RT-PCR during kiwifruit development and ripening. Different letters refers to significant differences between the samples according to Student’s t-test (a,b *p* < 0.01; b,c *p* < 0.05). daa = days after anthesis; MNE = mean normalized expression.

Having confirmed the activity of the AdTDC enzyme, we carried out quantitative RT-PCR analysis to monitor the expression of the *AdTDC* gene during kiwifruit development (Figure 12). The highest expression level was detected at the onset of fruit formation, our earliest sampling point (24 daa). The abundance of *AdTDC* mRNA fell by 10-fold (*p* < 0.01) by the second sampling point (51 daa) and remained at this level until 101 dpa. A further reduction (*p* < 0.05) was observed at the later ripening phase (122 and 142 dpa). Moreover, a low level of expression was maintained with no significant differences in the fully-ripe fruits in both 2014 and 2015. This expression profile, and in particular the high level of expression observed at the beginning of fruit formation and development, indicates that TDC and the associated metabolites (tryptamine and serotonin) play a key role in kiwifruit development, particularly at the early developmental stages.

## 3. Discussion

*Actinidia deliciosa* cv Hayward fruits were collected at different time points after anthesis in order to investigate the dynamic developmental profile of primary and secondary metabolites. Kiwifruit berries present three different phases from anthesis to fully-ripe fruits: expansion, ripening and senescence. Fruit ripening is preceded by the “mature fruit” stage at 140–160 daa [38,39]. Here, we collected fruits up to 142 daa, thus considering only fruits during and at the end of the expansion phase. Before starting the metabolic profiling, we measured agronomic characteristics such as weight and degrees of Brix, and these were in line with previous reports describing the same species [40].

### 3.1. Untargeted Metabolomics Provides Novel Markers of Fruit Development

Untargeted metabolomics analysis (high-resolution UPLC-MS) revealed the presence of many secondary metabolites in developing fruits (flavonoids, chalcones, coumarins, hydroxycinnamic and hydroxybenzoic acid derivatives, and procyanidins) as well as bound aroma precursors (volatiles), some lipids and some organic acids. Although kiwifruits are widely consumed, most articles that describe their secondary metabolites have focused only on the major compounds [41,42,43] or small sets thereof [44], rather than comprehensive analysis. The dataset presented herein includes 81 different metabolites, which is the most extensive collection of tentatively annotated secondary metabolites described thus far for fully-ripe kiwifruits. We also charted the dynamic behavior of these metabolites during development.

The changing abundance of metabolites during fruit development reflects a combination of de novo synthesis, consumption, interconversion, modification, and also dilution factors caused by cell expansion. The growth phase of *Actinidia deliciosa* cv Hayward fruits is well characterized [16,45,46,47]. The fruits reach their final size ~150 daa. This follows an initial rapid growth phase lasting ~50 days, which is characterized by a large gain in fresh weight but only a small gain in dry weight, with low levels of sugar and starch, followed by a slower growth phase that lasts ~100 days, characterized by an increase in dry weight, the accumulation of starch, and then the accumulation of sugar as starch accumulation slows [16]. We found that the most profound metabolic changes occurred during the initial rapid growth phase, including the depletion of flavonoids, chalcones, hydroxybenzoic acid derivatives, procyanidins, lipids and bound volatiles, and the accumulation or depletion of hydroxycinnamic acid derivatives. The declining levels of most secondary metabolites during this phase probably reflected the allocation of resources to support growth, as also indicated by the concomitant depletion of sucrose.

During the slower growth phase, the flavonoid content continued to decrease, alongside the slowing accumulation of starch and the beginning of free sucrose accumulation [48]. Kiwifruit development as a whole therefore appears to involve the steady depletion of most phenolic compounds both as a proportion of fruit weight and in terms of the absolute content per fruit. This indicates that metabolite depletion is not solely due to the dilution effect caused by cell expansion but is a genuine metabolic phenomenon, although many phenolic compounds were still detected in the fully-ripe fruits. In contrast, we detected two coumarins and several hydroxycinnamic acids that accumulated during development, suggesting a role in the ripening process. Of particular interest, we detected a number of bound volatiles that are known precursors of kiwifruit aroma compounds, including linalool, geraniol, nerol, phenylethanol, and hexanol [49,50].

### 3.2. ABA is Involved in Kiwifruit Development

ABA is a phytohormone involved in stress responses, the promotion of seedling growth and seed dormancy [51,52,53]. ABA participates in the development and ripening of both climacteric and non-climacteric fruits [31,32]. For example, ABA-deficient mutants of the climacteric model species tomato produced smaller fruits [54,55], whereas silencing an ABA receptor gene (*FaPYR1*) in the non-climacteric model species strawberry inhibited fruit development and delayed ripening [31]. Strawberry ripening was also delayed by mutating the *FaNCED1* gene, which is required for ABA biosynthesis, resulting in pale fruits with low levels of this hormone [56]. Exogenous ABA enhances the accumulation of sugars in grapevine berries [57] and developing melons [58]. Here, we detected low levels of ABA in kiwifruits at the first sampling stage (24 daa) and the ABA content increased thereafter, reaching a peak at 101 daa. Accordingly, Li et al. (2015) [39] found that the *Achn379301* gene, encoding the aldehyde oxidase that converts ABA aldehyde into ABA, was most strongly expressed in *A. chinensis* fruits 100 daa. We found that the ABA levels in *A. deliciosa* started to decline after 122 daa, confirming the general trend observed for other climacteric fruits in which the depletion of ABA is often accompanied by a burst of ethylene production at the onset of ripening [59]. However, we also detected significant levels of ABA during the final developmental stage and in the fully-ripe fruits, in agreement with earlier reports showing that massive ethylene production in kiwifruits does not occur until senescence, and is associated with aroma compound accumulation and late softening [20,38]. The persistence of ABA in mature fruits suggests a role not only during development but also during ripening in this species, as is the case for non-climacteric fruits [60].

### 3.3. Tryptamine and Serotonin Levels during Development Reflect the Availability of TDC

Tryptamine and serotonin are tryptophan-derived indolamines that are found in many edible plants [61]. We detected high levels of both compounds in kiwifruits, in agreement with an earlier survey of various common fruits and vegetables [62]. Alongside kiwifruit, high levels of indolamines are also present in banana, pineapple, avocado, plum, cranberry, tomato, and some nuts of the family *Juglandaceae* [62,63]. Interestingly, serotonin has previously been shown to accumulate in a tissue-specific manner in banana fruits, initially increasing in the pulp of the green fruit as it ripens, but later accumulating in the over-ripe peel [64]. We found that tryptamine and serotonin were most abundant as a proportion of kiwifruit fresh weight at the earliest developmental time point we tested (24 daa) and then declined gradually as the fruit approached the mature stage (142 daa). Cell expansion and water accumulation during fruit development and ripening affect the concentration of metabolites so we also determined the quantity of both compounds per fruit. This revealed that both tryptamine and serotonin accumulated until 51 daa and then remained at steady levels through the rest of development. A different trend was reported for serotonin levels in pineapple, with a peak during the immature stage followed by a sharp decline in the ripe and over-ripe fruit [65]. Given that both serotonin and tryptamine accumulate to levels of tens of micrograms per gram fresh weight in various fruits, and that the process appears to be developmentally regulated, it is clear that both molecules are likely to fulfill important although as yet unknown functions in the developing fruits.

Tryptamine is synthesized by the enzyme TDC, a member of the type II pyridoxal phosphate-dependent aromatic amino acid decarboxylase family [66]. TDC requires pyridoxal 5′ phosphate for the decarboxylation reaction and the amino acid tryptophan is its substrate. The first plant *TDC* (from *C. roseus*) was characterized by De Luca et al. (1989) [29] and since then *TDCs* have been isolated from various species including *Ophiorrhiza pumila*, *Camptotheca acuminata*, *Capsicum annumm*, *Rauvolfia verticillata*, and *Oryza sativa* for functional expression in bacteria [35,67,68,69,70]. Given the high similarity between tryptophan and tyrosine decarboxylases, automated annotation is unreliable [71].

The presence of tryptamine and its direct derivative serotonin, produced by the action of tryptamine 5-hydroxylase [30], strongly suggested that genes coding for TDCs would be found in the kiwifruit genome. Accordingly, we identified putative *TDC* genes in both *A. deliciosa* and *A. chinensis*, and the enzymes were produced by transient expression in *N. benthamiana*, which does not produce tryptamine at detectable levels and thus provides a suitable expression background. Its close relative tobacco (*N. tabacum*) was previously chosen for a similar purpose due to the lack of significant TDC activity [72]. *A. chinensis TDC* candidate genes were previously selected by screening kiwifruit genetic resources using BlastP and *C. roseus* TDC as the query [34]. The conversion of tryptophan to tryptamine by TDC occurs in the cytosol of *C. roseus* cells [73,74] and all three putative *A. chinensis* TDCs were predicted to be cytosolic. We selected Achn173261 because it was the most similar to CrTDC, and we cloned the coding sequence to verify its activity in vivo.

By using the *A. chinensis TDC* gene described above, we isolated 20 candidate *A. deliciosa TDCs*, and the one with the highest similarity to *A. chinensis* was analyzed in more detail. The alignment of the deduced Achn173261, AdTDC11, PepTDC1 and CrTDC amino acid sequences revealed that both *Actinidia* TDCs shared many conserved regions and typical TDC enzymatic features [36]. The construction of a phylogenetic tree including already characterized TDCs from other plant species confirmed that Achn173261 and AdTDC11 belong to the TDC clade.

The transient expression of *Achn173261* and *AdTDC11* in *N. benthamiana* leaves followed by HPLC-ESI/MS analysis revealed that both candidate genes encoded TDCs that were able to convert tryptophan into tryptamine in vivo. Despite the 99.2% identity between the two deduced amino acid sequences (only four mismatching amino acid residues), the expression of *AdTDC11* resulted in the production of more tryptamine in *N. benthamiana* leaves than the expression of *Achn173261*. We speculate that this difference in performance may reflect the effect of the four distinct residues on the folding of the polypeptide backbone in the area of the substrate-binding and/or catalytic domains [75].

The analysis of *AdTDC* expression revealed that the mRNA level was very high at the first sampling stage but decreased sharply thereafter. Similarly, high levels of tryptamine were detected at the beginning of development, but the subsequent reduction was more gradual. This suggests that kiwifruit cells consume tryptamine very slowly or that the enzyme is very stable, resulting in a lag between the decline in mRNA levels and the abundance of the corresponding protein. It is also possible that other, yet uncharacterized, *TDC* genes are involved in this physiological process.

## 4. Materials and Methods

### 4.1. Sampling Plan

*Actinidia deliciosa* cv. Hayward fruits were collected 24, 51, 75, 101, 122, and 142 daa (29 May, 2016) from an orchard located in Pescantina, Verona, Italy (GPS: 45.485749, 10.826099). In more detail, 60 kiwifruits were collected from five different plants widely distributed along two rows. Fruits were put in paper bags, transferred to the laboratory and processed within one hour. Fruits were assigned to three groups of 20, which were immediately weighed and peeled. Fruits were cut longitudinally into eight slices and one was immediately frozen and powdered in liquid nitrogen using an A11 analytical mill (IKA-Werke, Staufen, Germany). The rest were used to measure the degrees Brix with a DBR 35 digital refractometer (Ningbo Kingstic, Ningbo, China). Fully-ripe fruits (Consorzio Ortofrutticolo Padano) were provided on 26 November 2014 and 2 April 2015 and assigned to three pools of 50 for metabolomics analysis and another 30 fruits were used to measure the degrees Brix as above. A radial section of each ripe fruit was prepared using a cutter to ensure that all fruit tissues were represented. The pieces were immediately frozen and powdered with liquid nitrogen as above. Fruit powders were stored at −80 °C.

### 4.2. Metabolite Extraction for Targeted and Untargeted UPLC-MS Analysis

We extracted 150 mg of frozen powder in 450 µL of LC-MS grade methanol (Honeywell, Seezle, Germany). The mixture was vortexed for 30 s and sonicated at 40 kHz in a Sonica Ultrasonic Cleaner ultrasonic bath (SOLTEC, Milano, Italy) for 10 min before two rounds of centrifugation at 14,000× *g* for 15 min each. For the untargeted metabolomics analysis, the methanol extracts were diluted 1:3 (*v*/*v*) with LC-MS grade water (Honeywell), passed through Minisart RC4 filters with 0.2 µm pores (Sartorius, Göttingen, Germany) and 5 µL was injected into the UPLC device. For targeted metabolomics analysis, tryptamine and serotonin were quantified by diluting the samples 1:20 (*v*/*v*) with LC-MS grade water including the authentic commercial standards d_4_-tryptamine and d_4_-serotonin (Sigma, Darmstadt, Germany). The hydro-alcoholic mixtures containing the deuterated molecules at final concentrations of 5 pg/µL were passed through Minisart RC4 filters (0.2 µm pores) and 1 µL was injected into the UPLC device.

### 4.3. ABA Extraction for Targeted UPLC-MS Analysis

ABA levels were determined as previously described [33] with slight modifications. Briefly, 300 mg of frozen powder was extracted with 2 mL of cold methanol/water (80:20, *v*/*v*) including 20 mg/mL of butylated hydroxytoluene (Sigma). The extracts were vortexed for 10 s and continuously shaken on a 205F ROT rotary shaker (FALC) at 4 °C for 16 h in the dark. The samples were centrifuged twice at 14,000× *g* for 15 min at 4 °C. The extracts were diluted 1:3 (*v*/*v*) with LC-MS grade water including d_6_-abscisic acid (Olchemim, Olomouc, Czech Republic) at a final concentration of 1 pg/µL and passed through Minisart RC4 filters (0.2 µm pores). Finally, 5 µL was injected to the UPLC device.

### 4.4. UPLC-MS Conditions for Targeted and Untargeted Metabolomics Analysis

An ACQUITY I CLASS UPLC system (Waters, Milford, MA, USA) was connected to a Xevo G2-XS qTOF mass spectrometer (Waters) featuring an electrospray ionization (ESI) source operating in either positive or negative ionization mode and was controlled by MassLynx v4.1. All extracts were injected into a Waters ACQUITY UPLC BEH C18 column (2.1 mm × 100 mm, 1.7 μm) kept at 30 °C and the mobile phases consisted of 0.1% formic acid in water (A) and acetonitrile (B). The initial conditions were 99% A and 1% B, and the following elution profile was applied: 0–1 min, 1% B; 1–10 min, 1–40% B; 10–13.50 min, 40–70% B; 13.50–14.00 min, 70–99% B; 14.00–16.00 min, 99% B; 16.00–16.10 min, 99–1% B (initial conditions). Subsequently, the system was equilibrated in 99% A and the elution was complete after 20 min. The flow rate was set to 0.350 mL/min. Samples were kept at 8 °C and randomized. A quality control (QC) sample was prepared by mixing equal parts of 24, 75, and 142 daa methanol extracts in order to check the UPLC-qTOF performance along the whole experiment. QC was injected after nine samples had been analyzed. The ion source parameters were: capillary voltage 0.8 kV, sampling cone voltage 40 V, source offset voltage 80 V, source temperature 120 °C, desolvation temperature 500 °C, cone gas flow rate 50 L/h and desolvation gas flow rate 1000 L/h. Nitrogen gas was used for the nebulizer and in desolvation whereas argon was used to induce collision-induced dissociation. An MS method was created to acquire data in continuum mode using a fixed collision energy in two scan functions. In function 1, the low energy was disabled, whereas in function 2 the high energy was set to 35 V. For some samples, the high energy was increased to 45 V in order to achieve the better fragmentation of certain metabolites. In both functions, the Xevo G2-XS was set to perform the analysis in sensitivity mode, within the range 50–2000 *m*/*z* and with a scan time of 0.3 s. The lock mass solution used as “calibrator” to verify the accuracy of the mass spectrometer consisted of a 100 pg/µL leucine-enkephalin solution (Waters) injected with a flow rate of 10 µL/min, generating a signal of 556.2771 in positive mode and 554.2615 in negative mode.

### 4.5. Data Processing and Metabolite Identification

The raw data generated during the untargeted metabolomics analysis were processed using Progenesis QI software (Waters). An absolute ion intensity for peak picking was set at 10,000 and a minimum chromatographic peak width at 0.02 min. An automatic online search of public databases (MassBank, PlantCyc, Plant Metabolic Network and Human Metabolome Database) was used for tentative metabolite identification, by comparing *m*/*z* ratios, isotope similarities and fragmentation patterns, using the individual compound fragmentation extrapolated by Progenesis QI software. Further identifications were achieved using Metlin (https://metlin.scripps.edu) with a tolerance of 0.003 Da, and an in-house library of authentic standards. Finally, data reported in literature was used to support the putative annotations (for example, many dihydroferulic and dihydrocaffeic acid derivatives were observed in another work [76], as well). All the identifications were manually checked and in-source fragments were removed to avoid redundancy. The data matrix obtained by using Progenesis QI (Supplementary File 1), including *m*/*z* features and the relative abundances, was also submitted to principal component analysis (PCA) through SIMCA 13.0 (Umetrics) after Pareto scaling and centering.

For the targeted metabolomics analysis, MassLynx v4.1 (Waters) was used to manually extrapolate the peak areas relative to the tryptamine, serotonin and ABA signals and the corresponding deuterated commercially authentic standards. Peak extrapolation was based on the following *m*/*z* values: 160.0777 for serotonin and 164.1009 for d_4_-serotonin; 144.0863 for tryptamine and 148.1061 for d_4_-tryptamine; 263.1297 for ABA and 269.1671 for d_6_-ABA. For tryptamine and serotonin, the *m*/*z* values corresponded to the highest in-source generated fragment detected in positive ionization mode, whereas the ABA *m*/*z* feature corresponded to the molecular ion detected in negative ionization mode.

### 4.6. Analysis of TDC Sequences

To identify the *A. deliciosa* cv. Hayward TDC (AdTDC) amino acid sequence, the predicted *A. chinensis* proteome deposited in the Kiwifruit Genome Database (http://bioinfo.bti.cornell.edu) [34] was queried using the conserved amino acid sequence of CrTDC [29]. The resulting putative *A. chinensis* TDCs were submitted to InterProScan (https://www.ebi.ac.uk/interpro) to predict the family, domains and sites, and TargetP (http://www.cbs.dtu.dk/services/TargetP) was used to define the protein subcellular localization. The amino acid sequences of Achn173261, AdTDC11 (GenBank MK442011), PepTDC1 (GenBank ACN62127.1) and CrTDC (GenBank AAA33109.1) were aligned using Clustal Omega with default parameters (https://www.ebi.ac.uk/Tools/msa/clustalo/). MUSCLE alignment was carried out using the amino acid sequences of Achn107181, AdTDC, and other sequences reported by Hano et al. (2017) [37], except those derived from tomato. The final phylogenetic tree was generated using the neighbor-joining method and bootstrap analysis (1000 replicates) in MegaX software.

### 4.7. Gene Cloning

The coding sequence of *A. chinensis TDC* (*Achn173261*) was obtained by Geneart (Thermo Fisher Scientific, Bremen, Germany) and directionally cloned into the pENTR/D-TOPO vector (Invitrogen, Karlsruhe, Germany), yielding pENTR.Achn173261. Total RNA was extracted from 100 mg frozen powder (prepared from fruits collected in 2014) using the Spectrum Plant Total RNA Kit (Sigma), followed by DNase treatment with Turbo DNase (Invitrogen) according to the manufacturers’ instructions. SuperScriptIII Reverse Transcriptase (Invitrogen) was used to prepare cDNA from 2 µg total RNA from each sample. Primers specific for the coding sequence of *AdTDC* (Table S1 in Supplementary File 2) were used to amplify total cDNA using the high-fidelity KAPA HiFi DNA polymerase (KAPABIOSYSTEM, Roche, Basel, Switzerland) and primers flanking the *Achn173261* coding sequence. The PCR products were directionally cloned into pENTR/D-TOPO and 20 clones were sequenced. The amplified sequence with the highest similarity to *Achn173261*, together with pENTR.Achn173261, was transferred to the binary overexpression vector pK7WG2 by LR recombination [77].

### 4.8. Transient Expression and HPLC-ESI/MS Analysis

*Nicotiana benthamiana* plants were used for transient expression as described by Avesani et al. (2014) [78]. Briefly, pK7WG2.Achn173261 and pK7WG2.AdTDC11 were introduced into *A. tumefaciens* strain EHA105 as well as the negative control pK7WG2 harboring the *gfp* marker gene. Three leaves per 5-week-old *N. benthamiana* plant were syringe infiltrated with a bacterial suspension (OD_600_ of 0.9). Three plants were treated with each construct and one leaf (a biological replicate) was collected from each plant after 3 days, then another after 4 days and another after 5 days. The leaves were immediately homogenized in liquid nitrogen and the metabolites were extracted as described above. The presence of tryptamine was determined by HPLC-ESI/MS as follows. The instrument setup consisted of a Gold 508 Autosampler (Beckman Coulter) set at 4 °C in front of a Gold 127 HPLC system (Beckman Coulter) equipped with a reversed phase (RP) Alltima HP C18 column (150 × 2.1 mm, particle size 3 μm) protected with a C18 guard column (7.5 × 2.1 mm, particle size 5 μm). The flow rate was set at 0.2 mL/min and the two mobile phases consisted of 0.05% formic acid in water (A) and 0.05% formic acid in acetonitrile (B). The initial conditions were 98% A and 2% B, and the following elution profile was applied: 0–2 min, 2% B; 2–10 min, 2–10% B; 10–20 min, 10–60% B; 20–30 min, 60–90% B; 30–35 min, 90% B; 35–36 min, 2–90% B (initial conditions). Subsequently, the system was equilibrated in 98% A and the method was complete in 60 min. Samples were randomized and 20 µL was injected. The metabolites were detected using an Esquire 6000 ion trap mass spectrometer (Bruker Daltonics GmbH, Bremen, Germany) performing a scan range of 50–400 *m*/*z* with a target mass of 200 *m*/*z*. The ESI source operated in alternate positive mode, exploiting nitrogen as both nebulizing (50 psi and 350 °C) and drying gas (10 L/min). The chromatographic data files were recorded with the Esquire v5.2 Control software (Bruker Daltonik GmbH, Bremen, Germany) and the generated mass spectra data files were processed using Data Analysis v3.2 software (Bruker Daltonik GmbH).

### 4.9. AdTDC Expression Analysis

Total RNA from 150 mg of tissue was extracted, processed and reverse transcribed as specified above. The synthesized cDNA served as a template for quantitative real-time PCR analysis to assess the expression levels of the *AdTDC* gene. The kiwifruit actin gene (*Achn107181*, Kiwifruit Genome Database), whose constitutive expression was checked in all the samples prior to analysis, was chosen as the reference gene. The primers used to generate amplicons 90–100 bp from the coding region are listed in Table S1 of Supplementary File 2. Given that few differences were found in the nucleotide sequences of the 20 *AdTDC* clones, the PCR primers were designed to anneal at a conserved region allowing the amplification of all the putative *TDC* genes. The cDNA samples were diluted 1:10 with nuclease-free water, and a 25-µL reaction was performed in triplicate for each biological replicate using the GoTaq qPCR Master Mix (Promega, Madison, WI, USA) on a StepOne Plus instrument (Applied Biosystems, Foster City, CA, USA). Raw data were processed with LinRegPCR software and the *AdTDC* transcript levels were expressed as MNE by relative Ct comparison with the reference gene.

## 5. Conclusions

The molecular basis of fruit development and ripening is well characterized in model species, but little information is available for non-model species such as kiwifruit. Here we used a combination of high-resolution targeted and untargeted metabolomics analysis to provide a broad picture of the dynamic metabolic profile of kiwifruits during development and ripening, focusing on secondary metabolites, which are responsible for economically important flavor and aroma characteristics. We found that fruit development involves the depletion of many phenolic compounds (hydroxybenzoic acids, flavonoids, flavan-3-ols, chalcones and procyanidins), as well as lipids and bound-volatiles, but some metabolites became more abundant, including coumarins and some hydroxycinnamic acids. The accumulation of ABA indicated that this phytohormone plays a key role in both development and ripening. Finally, the high content of tryptamine and serotonin in developing kiwifruits, together with the burst of *TDC* expression early in development, provides strong evidence that indolamines fulfill an unrecognized function in developing kiwifruits. Our data provide the basis for further, more detailed analysis of the dynamic metabolism of developing kiwifruits, including the precise functions of ABA and indolamines. The investigation of the biological roles of tryptamine and serotonin in fleshy fruits of a non-model species, such as in kiwifruit, is difficult due to the lack of protocols and widely annotated genetic resources. Therefore, the characterization of *TDCs* in a plant model species, i.e., the *Solanum lycopersicum* cv. Microtom, able to accumulate tryptamine and serotonin in fleshy fruits, is currently under investigations.

## Figures and Tables

**Table 1:** Putative orthologs of *Catharanthus roseus* tryptophan decarboxylase in *Actinidia chinensis*. CrTDC is ~500 amino acids (aa) in length.

Sequence	Length (aa)	Identity (%)	Positives (%)	Score (bits)	TargetP ^1^
Achn173261	501	65	80	689	Other (0.869)
Achn127711	548	52	72	568	Other (0.833)
Achn173271	483	50	72	483	Other (0.686)

^1^ TargetP numbers indicate scores for cellular location.

## Supplementary Materials

Supplementary materials can be found at http://www.mdpi.com/1422-0067/20/4/897/s1. Supplementary File 1: Untargeted metabolomics data matrix; Supplementary File 2 includes: Table S1: Primer list; Figure S1: ABA peak detected by negative UPLC-MS analysis; Figure S2: ClustalW alignment of the amino acid sequences of AdTDC11 and Achn173261; Figure S3: Clustal Omega alignment of the candidate *Actinidia deliciosa TDCs*.

## Abbreviations

TDC	Tryptophan decarboxylase
Daa	Days after anthesis
UPLC-qTOF	Ultra Performance Liquid Chromatography-quadrupole Time of Flight
HPLC-ESI/MS	High Performance Liquid Chromatography-Electrospray Ionization/Mass Spectrometry
