# Supplementary material for: Untargeted and Targeted Metabolomics and Tryptophan Decarboxylase In Vivo Characterization Provide Novel Insight on the Development of Kiwifruits (Actinidia deliciosa)
Source: Int J Mol Sci. 2019 Feb 19;20(4):897. doi: 10.3390/ijms20040897 (PMC6413197; doi:10.3390/ijms20040897)
Supplement: Supplementary file 1 [file ijms-20-00897-s001.zip › ijms-446273 Supplementary Files-final check version/Supplementary File 2.pdf]

International Journal of
Molecular Sciences

MDPI

Untargeted and targeted metabolomics and Tryptophan Decarboxylase *in vivo* characterization provide novel insight on the development of kiwifruits (*Actinidia deliciosa*)

Supplementary File 2

Table 1. Primer list.

Gene description	Primer direction	Primer length (bp)	Tm (°C)	Amplicon length (bp)	Primer Sequence
AdTDC	FOR	22	61*	1510	**<u>CACC</u>**ATGGGCAGCCTCGACTCC
	REV	25	61*		CTATGTAGCATCCTTTGACCTGAGC
AdTDC (q-RT-PCR)	FOR	20	65	98	TGGCAAGTGGGTACGGGTCG
	REV	20	65		TCGGACCGGATGTGGCTCTG
Actin (q-RT-PCR; Achn107181)	FOR	20	65	94	GTCTCGACCTTGCTGGCCGT
	REV	20	65		ATTTCCCGTTCGGCCGTGGT

The CACC tag for the directional cloning is underlined in bold. Asterisks indicate the Tm assessed by OligoPerfect (Thermo Fisher Scientific).

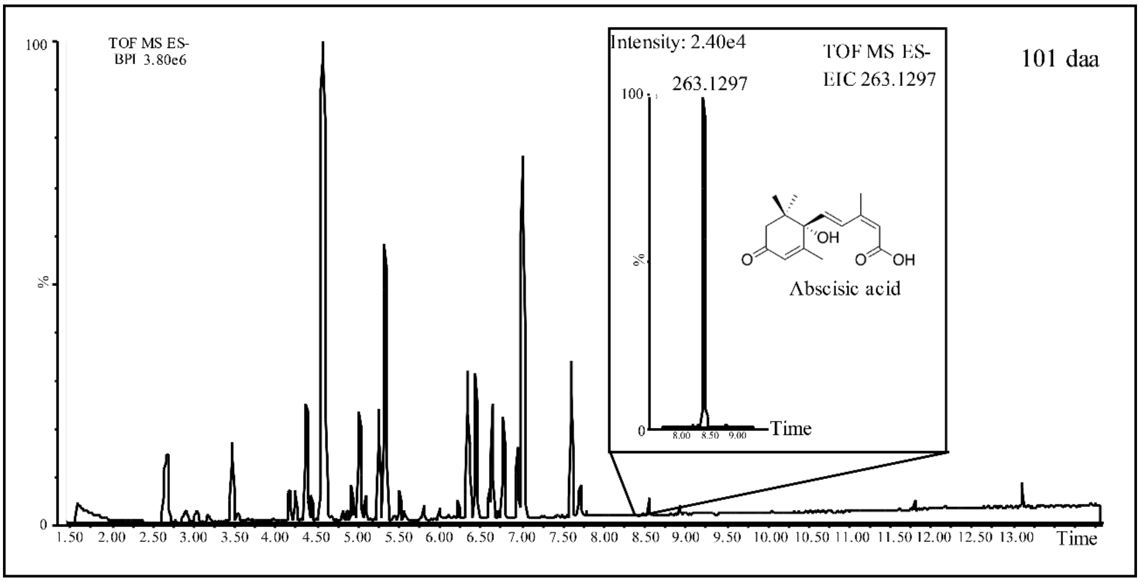

Figure S1: ABA peak detected by negative UPLC-MS analysis. The sample is a kiwifruit methanol extract corresponding to 101 days after anthesis (daa). The box highlights the peak corresponding to abscisic acid.

```
# Aligned_sequences: 2
# 1: Achn173261
# 2: AdTDC11
# Matrix: EBLOSUM62
# Gap_penalty: 10.0
# Extend_penalty: 0.5
#
# Length: 501
# Identity:     497/501 (99.2%)
# Similarity:   499/501 (99.6%)
# Gaps:           0/501 ( 0.0%)
# Score: 2635.0
#
#
#=======================================

Achn173261         1 MGSLDSNFSVNSTEFKPLDPEEFRKQAHTMVDFIADYYKNIETYPVLSQA     50
                     ||||||||||||||||||||||||||||||||||||||||||||||||||
AdTDC11            1 MGSLDSNFSVNSTEFKPLDPEEFRKQAHTMVDFIADYYKNIETYPVLSQA     50

Achn173261        51 QPGYLRARLPETAPYRPETFETILQDVEKSIIPGITHWLSPKFFAFFPAT    100
                     ||||||||||||||||||||||||||||||||||||||||||||||||||
AdTDC11           51 QPGYLRARLPETAPYRPETFETILQDVEKSIIPGITHWLSPKFFAFFPAT    100

Achn173261       101 VSTAAFLGEMLCTCFNSVGFNWLASPAATELEMVVMDWLAHMLKLPKSFM    150
                     ||||||||||||||||||||||||||||||||||||||||||||||||||
AdTDC11          101 VSTAAFLGEMLCTCFNSVGFNWLASPAATELEMVVMDWLAHMLKLPKSFM    150

Achn173261       151 FQGTGGGVIQGTTSDSILCTLVAARNRALENVGVDNIGKLVVYGSDQTHS    200
                     |||||||||||||||||||||||||||.||||||||||||||||||||||
AdTDC11          151 FQGTGGGVIQGTTSDSILCTLVAARNRTLENVGVDNIGKLVVYGSDQTHS    200

Achn173261       201 TYTKACKLAGIYPRNIRSVPAGSDTCFAMSPVALRKVIDADLAAGMVPLY    250
                     |||||||||||||||||||||||||||||||||||||||||||||:||||
AdTDC11          201 TYTKACKLAGIYPRNIRSVPAGSDTCFAMSPVALRKVIDADLAAGLVPLY    250

Achn173261       251 LCVTVGTTSTTAVDPLEDLADVANDYGVWVHVDAAYGGSACICPEFRHYL    300
                     ||||||||||||||||||||||||||||||||||||||||||||||||||
AdTDC11          251 LCVTVGTTSTTAVDPLEDLADVANDYGVWVHVDAAYGGSACICPEFRHYL    300

Achn173261       301 DGIELVDSLSLSPHKWLLSYLDCCCLWVKQPGLLVKALSTNPEYLRNKPR    350
                     |||||||||||||||||||||||||||||||||||||||||||||||||.
AdTDC11          301 DGIELVDSLSLSPHKWLLSYLDCCCLWVKQPGLLVKALSTNPEYLRNKPS    350

Achn173261       351 ESDSVVDYKDWQVGTGRRFKALRLWLVLRSYGIANLQSHIRSDVRMAKMF    400
                     ||||||||||||||||||||||||||||||||||||||||||||||||||
AdTDC11          351 ESDSVVDYKDWQVGTGRRFKALRLWLVLRSYGIANLQSHIRSDVRMAKMF    400

Achn173261       401 EGFVKSDPRFELVVPRIFSLVCFRLNPHPGSYPGYLELLNKKLLEWVNST    450
                     ||||||||||||||||||||||||||||||||||||||||||||||||||
AdTDC11          401 EGFVKSDPRFELVVPRIFSLVCFRLNPHPGSYPGYLELLNKKLLEWVNST    450

Achn173261       451 GGLYMTHTVAGGLYMLRFAVGATLTEECHVVAAWKIIKEGADVLLRSKDA    500
                     |||||||||||||||||||||||||||||||||||:||||||||||||||
AdTDC11          451 GGLYMTHTVAGGLYMLRFAVGATLTEECHVVAAWKLIKEGADVLLRSKDA    500

Achn173261       501 T    501
                     |
AdTDC11          501 T    501
```

Figure S2: ClustalW alignment of the amino acid sequences of AdTDC11 and Achn173261.

```
Achn173261   ATGGGCAGCCTCGACTCCAACTTCTCCGTCAACTCCACCGAATTCAAACCGCTCGACCCC   60
AdTDC1       ATGGGCAGCCTCGACTCCAACTTCTCCGTCAACTCCACCGAATTCAAACCGCTCGACCCC   60
AdTDC2       ATGGGCAGCCTCGACTCCAACTTCTCCGTCAACTCCACCGAATTCAAACCGCTCGACCCC   60
AdTDC3       ATGGGCAGCCTCGACTCCAACTTCTCCGTCAACTCCACCGAATTCAAACCGCTCGACCCC   60
AdTDC4       ATGGGCAGCCTCGACTCCAACTTCTCCGTCAACTCCACCGAATTCAAACCGCTCGACCCC   60
AdTDC5       ATGGGCAGCCTCGACTCCAACTTCTCCGTCAACTCCACCGAATTCAAACCGCTCGACCCC   60
AdTDC6       ATGGGCAGCCTCGACTCCAACTTCTCCGTCAACTCCACCGAATTCAAACCGCTCGACCCC   60
AdTDC7       ATGGGCAGCCTCGACTCCAACTTCTCCGTCAACTCCACCGAATTCAAACCGCTCGACCCC   60
AdTDC8       ATGGGCAGCCTCGACTCCAACTTCTCCGTCAACTCCACCGAATTCAAACCGCTCGACCCC   60
AdTDC9       ATGGGCAGCCTCGACTCCAACTTCTCCGTCAACTCCACCGAATTCAAACCGCTCGACCCC   60
AdTDC10      ATGGGCAGCCTCGACTCCAACTTCTCCGTCAACTCCACCGAATTCAAACCGCTCGACCCC   60
AdTDC11      ATGGGCAGCCTCGACTCCAACTTCTCCGTCAACTCCACCGAATTCAAACCGCTCGACCCC   60
AdTDC12      ATGGGCAGCCTCGACTCCAACTTCTCCGTCAACTCCACCGAATTCAAACCGCTCGACCCC   60
AdTDC13      ATGGGCAGCCTCGACTCCAACTTCTCCGTCAACTCCACCGAATTCAAACCGCTCGACCCC   60
AdTDC14      ATGGGCAGCCTCGACTCCAACTTCTCCGTCAACTCCACCGAATTCAAACCGCTCGACCCC   60
AdTDC15      ATGGGCAGCCTCGACTCCAACTTCTCCGTCAACTCCACCGAATTCAAACCGCTCGACCCC   60
AdTDC16      ATGGGCAGCCTCGACTCCAACTTCTCCGTCAACTCCACCGAATTCAAACCGCTCGACCCC   60
AdTDC17      ATGGGCAGCCTCGACTCCAACTTCTCCGTCAACTCCACCGAATTCAAACCGCTCGACCCC   60
AdTDC18      ATGGGCAGCCTCGACTCCAACTTCTCCGTCAACTCCACCGAATTCAAACCGCTCGACCCC   60
AdTDC19      ATGGGCAGCCTCGACTCCAACTTCTCCGTCAACTCCACCGAATTCAAACCGCTCGACCCC   60
AdTDC20      ATGGGCAGCCTCGACTCCAACTTCTCCGTCAACTCCACCGAATTCAAACCGCTCGACCCC   60
             ************************************************************

Achn173261   GAAGAGTTCCGAAAACAAGCCCATACCATGGTAGATTTCATTGCAGACTACTACAAGAAC   120
AdTDC1       GAAGAGTTCCGTAAACAAGCCCATACCATGGTAGATTTCATTGCAGACTACTACAAGAAC   120
AdTDC2       GAAGAGTTCCGTAAACAAGCCCATACCATGGTAGATTTCATTGCAGACTACTACAAGAAC   120
AdTDC3       GAAGAGTTCCGTAAACAAGCCCATACCATGGTAGATTTCATTGCGGACTACTACAAGAAC   120
AdTDC4       GAAGAGTTCCGTAAACAAGCCCATACCATGGTAGATTTCATTGCGGACTACTACAAGAAC   120
AdTDC5       GAAGAGTTCCGTAAACAAGCCCATACCATGGTAGATTTCATTGCAGACTACTACAAGAAC   120
AdTDC6       GAAGAGTTCCGTAAACAAGCCCATACCATGGTAGATTTCATTGCAGACTACTACAAGAAC   120
AdTDC7       GAAGAGTTCCGTAAACAAGCCCATACCATGGTAGATTTCATTGCGGACTACTACAAGAAC   120
AdTDC8       GAAGAGTTCCGTAAACAAGCCCATACCATGGTAGATTTCATTGCAGACTACTACAAGAAC   120
AdTDC9       GAAGAGTTCCGAAAACAAGCCCATACCATGGTAGATTTCATTGCAGACTACTACAAGAAC   120
AdTDC10      GAAGAGTTCCGAAAACAAGCCCATACCATGGTAGATTTCATTGCAGACTACTACAAGAAC   120
AdTDC11      GAAGAGTTCCGAAAACAAGCCCATACCATGGTAGATTTCATTGCAGACTACTACAAGAAC   120
AdTDC12      GAAGAGTTCCGAAAACAAGCCCATACCATGGTAGATTTCATTGCAGACTACTACAAGAAC   120
AdTDC13      GAAGAGTTCCGAAAACAAGCCCATACCATGGTAGATTTCATTGCAGACTACTACAAGAAC   120
AdTDC14      GAAGAGTTCCGAAAACAAGCCCATACCATGGTAGATTTCATTGCAGACTACTACAAGAAC   120
AdTDC15      GAAGAGTTCCGAAAACAAGCCCATACCATGGTAGATTTCATTGCAGACTACTACAAGAAC   120
AdTDC16      GAAGAGTTCCGAAAACAAGCCCATACCATGGTAGATTTCATTGCAGACTACTACAAGAAC   120
AdTDC17      GAAGAGTTCCGAAAACAAGCCCATACCATGGTAGATTTCATTGCAGACTACTACAAGAAC   120
AdTDC18      GAAGAGTTCCGAAAACAAGCCCATACCATGGTAGATTTCATTGCAGACTACTACAAGAAC   120
AdTDC19      GAAGAGTTCCGAAAACAAGCCCATACCATGGTAGATTTCATTGCAGACTACTACAAGAAC   120
AdTDC20      GAAGAGTTCCGAAAACAAGCCCATACCATGGTAGATTTCATTGCAGACTACTACAAGAAC   120
```

```
               *********** ******************************** ***************

Achn173261     ATCGAAACCTACCCGGTTCTCAGCCAAGCCCAACCGGGCTACCTACGGGCCCGCTTACCC    180
AdTDC1         ATCGAAACCTACCCGGTTCTCAGCCAAGCCCAACCGGGCTACCTACGGGCACGCTTACCC    180
AdTDC2         ATCGAAACCTACCCGGTTCTCAGCCAAGCCCAACCGGGCTACCTACGGGCACGCTTACCC    180
AdTDC3         ATCGAAACCTACCCGGTTCTCAGCCAAGCCCAACCAGGCTACCTACGGGCACGCTTACCC    180
AdTDC4         ATCGAAACCTACCCGGTTCTCAGCCAAGCCCAACCAGGCTACCTACGGGCACGCTTACCC    180
AdTDC5         ATCGAAACCTACCCGGTTCTCAGCCAAGCCCAACCGGGCTACCTACGGGCACGCTTACCC    180
AdTDC6         ATCGAAACCTACCCGGTTCTCAGCCAAGCCCAACCGGGCTACCTACGGGCACGCTTACCC    180
AdTDC7         ATCGAAACCTACCCGGTTCTCAGCCAAGCCCAACCAGGCTACCTACGGGCACGCTTACCC    180
AdTDC8         ATCGAAACCTACCCGGTTCTCAGCCAAGCCCAACCGGGCTACCTACGGGCACGCTTACCC    180
AdTDC9         ATCGAAACCTACCCGGTTCTCAGCCAAGCCCAACCGGGCTACCTACGGGCCCGCTTACCC    180
AdTDC10        ATCGAAACCTACCCGGTTCTCAGCCAAGCCCAACCGGGCTACCTACGGGCCCGCTTACCC    180
AdTDC11        ATCGAAACCTACCCGGTTCTCAGCCAAGCCCAACCGGGCTACCTACGGGCCCGCTTACCC    180
AdTDC12        ATCGAAACCTACCCGGTTCTCAGCCAAGCCCAACCGGGCTACCTACGGGCCCGCTTACCC    180
AdTDC13        ATCGAAACCTACCCGGTTCTCAGCCAAGCCCAACCGGGCTACCTACGGGCCCGCTTACCC    180
AdTDC14        ATCGAAACCTACCCGGTTCTCAGCCAAGCCCAACCGGGCTACCTACGGGCCCGCTTACCC    180
AdTDC15        ATCGAAACCTACCCGGTTCTCAGCCAAGCCCAACCGGGCTACCTACGGGCCCGCTTACCC    180
AdTDC16        ATCGAAACCTACCCGGTTCTCAGCCAAGCCCAACCGGGCTACCTACGGGCCCGCTTACCC    180
AdTDC17        ATCGAAACCTACCCGGTTCTCAGCCAAGCCCAACCGGGCTACCTACGGGCCCGCTTACCC    180
AdTDC18        ATCGAAACCTACCCGGTTCTCAGCCAAGCCCAACCGGGCTACCTACGGGCCCGCTTACCC    180
AdTDC19        ATCGAAACCTACCCGGTTCTCAGCCAAGCCCAACCGGGCTACCTACGGGCCCGCTTACCC    180
AdTDC20        ATCGAAACCTACCCGGTTCTCAGCCAAGCCCAACCGGGCTACCTACGGGCCCGCTTACCC    180
               *********************************** ************** *********

Achn173261     GAAACTGCCCCGTACCGCCCTGAAACGTTCGAAACCATACTACAAGACGTGGAGAAGAGC    240
AdTDC1         GAAACAGCCCCGTACCGCCCCGAAACGTTCGAAACCATACTACAAGACGTGGAGAAGAGC    240
AdTDC2         GAAACAGCCCCGTACCGCCCCGAAACGTTCGAAACCATACTACAAGACGTGGAGAAGAGC    240
AdTDC3         GAAACAGCCCCGTACCGCCCCGAAACGTTCGAAACCATACTACAAGACGTGGAGAAGAGC    240
AdTDC4         GAAACAGCCCCGTACCGCCCCGAAACGTTCGAAACCATACTACAAGACGTGGAGAAGAGC    240
AdTDC5         GAAACAGCCCCGTACCGCCCCGAAACGTTCGAAACCATACTACAAGACGTGGAGAAGAGC    240
AdTDC6         GAAACAGCCCCGTACCGCCCCGAAACGTTCGAAACCATACTACAAGACGTGGAGAAGAGC    240
AdTDC7         GAAACAGCCCCGTACCGCCCCGAAACGTTCGAAACCATACTACAAGACGTGGAGAAGAGC    240
AdTDC8         GAAACAGCCCCGTACCGCCCCGAAACGTTCGAAACCATACTACAAGACGTGGAGAAGAGC    240
AdTDC9         GAAACCGCCCCGTACCGCCCTGAAACGTTCGAAACCATACTACAAGACGTGGAGAAAAGC    240
AdTDC10        GAAACCGCCCCGTACCGCCCTGAAACGTTCGAAACCATACTACAAGACGTGGAGAAAAGC    240
AdTDC11        GAAACCGCCCCGTACCGCCCTGAAACGTTCGAAACCATACTACAAGACGTGGAGAAGAGC    240
AdTDC12        GAAACCGCCCCGTACCGCCCTGAAACGTTCGAAACCATACTACAAGACGTGGAGAAGAGC    240
AdTDC13        GAAACCGCCCCGTACCGCCCTGAAACGTTCGAAACCATACTACAAGACGTGGAGAAGAGC    240
AdTDC14        GAAACCGCCCCGTACCGCCCTGAAACGTTCGAAACCATACTACAAGACGTGGAGAAGAGC    240
AdTDC15        GAAACCGCCCCGTACCGCCCTGAAACGTTCGAAACCATACTACAAGACGTGGAGAAGAGC    240
AdTDC16        GAAACTGCCCCGTACCGCCCTGAAACGTTCGAAACCATACTACAAGACGTGGAGAAGAGC    240
AdTDC17        GAAACTGCCCCGTACCGCCCTGAAACGTTCGAAACCATACTACAAGACGTGGAGAAGAGC    240
AdTDC18        GAAACTGCCCCGTACCGCCCTGAAACGTTCGAAACCATACTACAAGACGTGGAGAAGAGC    240
```

```
AdTDC19      GAAACTGCCCCGTACCGCCCTGAAACGTTCGAAACCATACTACAAGACGTGGAGAAGAGC    240
AdTDC20      GAAACTGCCCCGTACCGCCCTGAAACGTTCGAAACCATACTACAAGACGTGGAGAAGAGC    240
             ***** ************** *********************************** ***

Achn173261   ATAATACCGGGAATCACCCATTGGCTGAGCCCTAAGTTTTTTGCTTTTTTTCCGGCTACC    300
AdTDC1       ATAATACCGGGAATCACCCATTGGCTGAGCCCTAAGTTTTTTGCTTTTTTTCCGGCCACC    300
AdTDC2       ATAATACCGGGAATCACCCATTGGCTGAGCCCTAAGTTTTTTGCTTTTTTTCCGGCCACC    300
AdTDC3       ATAATACCGGGAATCACCCATTGGCTGAGCCCTAAGTTTTTTGCTTTTTTTCCGGCCACC    300
AdTDC4       ATAATACCGGGAATCACCCATTGGCTGAGCCCTAAGTTTTTTGCTTTTTTTCCGGCCACC    300
AdTDC5       ATAATACCGGGAATCACCCATTGGCTGAGCCCTAAGTTTTTTGCTTTTTTTCCGGCCACC    300
AdTDC6       ATAATACCGGGAATCACCCATTGGCTGAGCCCTAAGTTTTTTGCTTTTTTTCCGGCCACC    300
AdTDC7       ATAATACCGGGAATCACCCATTGGCTGAGCCCTAAGTTTTTTGCTTTTTTTCCGGCCACC    300
AdTDC8       ATAATACCGGGAATCACCCATTGGCTGAGCCCTAAGTTTTTTGCTTTTTTTCCGGCCACC    300
AdTDC9       ATAATACCGGGAATCACCCATTGGCTGAGCCCTAAGTTTTTTGCTTTTTTTCCAGCCACC    300
AdTDC10      ATAATACCGGGAATCACCCATTGGCTGAGCCCTAAGTTTTTTGCTTTTTTTCCAGCCACC    300
AdTDC11      ATAATACCGGGAATCACCCATTGGCTGAGCCCTAAATTTTTTGCTTTTTTTCCGGCTACC    300
AdTDC12      ATAATACCGGGAATCACCCATTGGCTGAGCCCTAAATTTTTTGCTTTTTTTCCGGCTACC    300
AdTDC13      ATAATACCGGGAATCACCCATTGGCTGAGCCCTAAATTTTTTGCTTTTTTTCCGGCTACC    300
AdTDC14      ATAATACCGGGAATCACCCATTGGCTGAGCCCTAAATTTTTTGCTTTTTTTCCGGCTACC    300
AdTDC15      ATAATACCGGGAATCACCCATTGGCTGAGCCCTAAATTTTTTGCTTTTTTTCCGGCTACC    300
AdTDC16      ATAATACCGGGAATCACCCATTGGCTGAGCCCTAAGTTTTTTGCTTTTTTTCCGGCTACC    300
AdTDC17      ATAATACCGGGAATCACCCATTGGCTGAGCCCTAAGTTTTTTGCTTTTTTTCCGGCTACC    300
AdTDC18      ATAATACCGGGAATCACCCATTGGCTGAGCCCTAAGTTTTTTGCTTTTTTTCCGGCTACC    300
AdTDC19      ATAATACCGGGAATCACCCATTGGCTGAGCCCTAAGTTTTTTGCTTTTTTTCCGGCTACC    300
AdTDC20      ATAATACCGGGAATCACCCATTGGCTGAGCCCTAAGTTTTTTGCTTTTTTTCCGGCTACC    300
             *********************************** ***************** ** ***

Achn173261   GTGAGCACGGCGGCGTTTCTGGGGGAGATGCTGTGCACGTGCTTTAATTCTGTGGGGTTC    360
AdTDC1       GTGAGCACGGCGGCGTTTCTGGGGGAGATGCTGTGCACGTGCTTTAATTCTGTGGGGTTC    360
AdTDC2       GTGAGCACGGCGGCGTTTCTGGGGGAGATGCTGTGCACGTGCTTTAATTCTGTGGGGTTC    360
AdTDC3       GTGAGCACGGCGGCGTTTCTGGGGGAGATGCTGTGCACGTGCTTTAATTCTGTGGGGTTC    360
AdTDC4       GTGAGCACGGCGGCGTTTCTGGGGGAGATGCTGTGCACGTGCTTTAATTCTGTGGGGTTC    360
AdTDC5       GTGAGCACGGCGGCGTTTCTGGGGGAGATGCTGTGCACGTGCTTTAATTCTGTGGGGTTC    360
AdTDC6       GTGAGCACGGCGGCGTTTCTGGGGGAGATGCTGTGCACGTGCTTTAATTCTGTGGGGTTC    360
AdTDC7       GTGAGCACGGCGGCGTTTCTGGGGGAGATGCTGTGCACGTGCTTTAATTCTGTGGGGTTC    360
AdTDC8       GTGAGCACGGCGGCGTTTCTGGGGGAGATGCTGTGCACGTGCTTTAATTCTGTGGGGTTC    360
AdTDC9       GTGAGCACGGCGGCGTTTCTGGGGGAGATGCTGTGCACGTGCTTTAATTCTGTCGGGTTC    360
AdTDC10      GTGAGCACGGCGGCGTTTCTGGGGGAGATGCTGTGCACGTGCTTTAATTCTGTCGGGTTC    360
AdTDC11      GTGAGCACGGCGGCGTTTCTGGGGGAGATGCTGTGCACGTGCTTTAATTCTGTGGGGTTC    360
AdTDC12      GTGAGCACGGCGGCGTTTCTGGGGGAGATGCTGTGCACGTGCTTTAATTCTGTGGGGTTC    360
AdTDC13      GTGAGCACGGCGGCGTTTCTGGGGGAGATGCTGTGCACGTGCTTTAATTCTGTGGGGTTC    360
AdTDC14      GTGAGCACGGCGGCGTTTCTGGGGGAGATGCTGTGCACGTGCTTTAATTCTGTGGGGTTC    360
AdTDC15      GTGAGCACGGCGGCGTTTCTGGGGGAGATGCTGTGCACGTGCTTTAATTCTGTGGGGTTC    360
AdTDC16      GTGAGCACGGCGGCGTTTCTGGGGGAGATGCTGTGCACGTGCTTTAATTCTGTGGGGTTC    360
```

```
AdTDC17      GTGAGCACGGCGGCGTTTCTGGGGGAGATGCTGTGCACGTGCTTTAATTCTGTGGGGTTC   360
AdTDC18      GTGAGCACGGCGGCGTTTCTGGGGGAGATGCTGTGCACGTGCTTTAATTCTGTGGGGTTC   360
AdTDC19      GTGAGCACGGCGGCGTTTCTGGGGGAGATGCTGTGCACGTGCTTTAATTCTGTGGGGTTC   360
AdTDC20      GTGAGCACGGCGGCGTTTCTGGGGGAGATGCTGTGCACGTGCTTTAATTCTGTGGGGTTC   360
             ***************************************************** ******

Achn173261   AACTGGCTGGCCTCGCCGGCGGCAACGGAGCTGGAGATGGTGGTCATGGACTGGCTGGCC   420
AdTDC1       AACTGGCTGGCCTCGCCGGCGGCAACGGAGCTGGAGATGGTGGTCATGGACTGGCTGGCC   420
AdTDC2       AACTGGCTGGCCTCGCCGGCGGCAACGGAGCTGGAGATGGTGGTCATGGACTGGCTGGCC   420
AdTDC3       AACTGGCTGGCCTCGCCGGCGGCAACGGAGCTGGAGATGGTGGTCATGGACTGGCTGGCC   420
AdTDC4       AACTGGCTGGCCTCGCCGGCGGCAACGGAGCTGGAGATGGTGGTCATGGACTGGCTGGCC   420
AdTDC5       AACTGGCTGGCCTCGCCGGCGGCAACGGAGCTGGAGATGGTGGTCATGGACTGGCTGGCC   420
AdTDC6       AACTGGCTGGCCTCGCCGGCGGCAACGGAGCTGGAGATGGTGGTCATGGACTGGCTGGCC   420
AdTDC7       AACTGGCTGGCCTCGCCGGCGGCAACGGAGCTGGAGATGGTGGTCATGGACTGGCTGGCC   420
AdTDC8       AACTGGCTGGCCTCGCCGGCGGCAACGGAGCTGGAGATGGTGGTCATGGACTGGCTGGCC   420
AdTDC9       AACTGGTTGGCCTCGCCGGCGGCAACGGAGCTGGAGATGGTGGTCATGGACTGGCTGGCT   420
AdTDC10      AACTGGTTGGCCTCGCCGGCGGCAACGGAGCTGGAGATGGTGGTCATGGACTGGCTGGCT   420
AdTDC11      AACTGGCTGGCCTCGCCGGCGGCAACGGAGCTGGAGATGGTGGTCATGGACTGGCTGGCC   420
AdTDC12      AACTGGCTGGCCTCGCCGGCGGCAACGGAGCTGGAGATGGTGGTCATGGACTGGCTGGCC   420
AdTDC13      AACTGGCTGGCCTCGCCGGCGGCAACGGAGCTGGAGATGGTGGTCATGGACTGGCTGGCC   420
AdTDC14      AACTGGCTGGCCTCGCCGGCGGCAACGGAGCTGGAGATGGTGGTCATGGACTGGCTGGCC   420
AdTDC15      AACTGGCTGGCCTCGCCGGCGGCAACGGAGCTGGAGATGGTGGTCATGGACTGGCTGGCC   420
AdTDC16      AACTGGCTGGCCTCGCCGGCGGCAACGGAGCTGGAGATGGTGGTCATGGACTGGCTGGCC   420
AdTDC17      AACTGGCTGGCCTCGCCGGCGGCAACGGAGCTGGAGATGGTGGTCATGGACTGGCTGGCC   420
AdTDC18      AACTGGCTGGCCTCGCCGGCGGCAACGGAGCTGGAGATGGTGGTCATGGACTGGCTGGCC   420
AdTDC19      AACTGGCTGGCCTCGCCGGCGGCAACGGAGCTGGAGATGGTGGTCATGGACTGGCTGGCC   420
AdTDC20      AACTGGCTGGCCTCGCCGGCGGCAACGGAGCTGGAGATGGTGGTCATGGACTGGCTGGCC   420
             ****** ****************************************************

Achn173261   CACATGTTGAAGCTTCCTAAATCTTTCATGTTTCAAGGCACGGGTGGTGGGGTCATACAA   480
AdTDC1       CACATGTTGAAGCTTCCTAAATCTTTCATGTTTCAAGGCACTGGTGGTGGGGTCATACAA   480
AdTDC2       CACATGTTGAAGCTTCCTAAATCTTTCATGTTTCAAGGCACTGGTGGTGGGGTCATACAA   480
AdTDC3       CACATGTTGAAGCTTCCTAAATCTTTCATGTTTCAAGGCACTGGTGGTGGGGTCATACAA   480
AdTDC4       CACATGTTGAAGCTTCCTAAATCTTTCATGTTTCAAGGCACTGGTGGTGGGGTCATACAA   480
AdTDC5       CACATGTTGAAGCTTCCTAAATCTTTCATGTTTCAAGGCACTGGTGGTGGGGTCATACAA   480
AdTDC6       CACATGTTGAAGCTTCCTAAATCTTTCATGTTTCAAGGCACTGGTGGTGGGGTCATACAA   480
AdTDC7       CACATGTTGAAGCTTCCTAAATCTTTCATGTTTCAAGGCACTGGTGGTGGGGTCATACAA   480
AdTDC8       CACATGTTGAAGCTTCCTAAATCTTTCATGTTTCAAGGCACTGGTGGTGGGGTCATACAA   480
AdTDC9       CACATGTTGAAGCTTCCTAAATCTTTCATGTTTCAAGGCACTGGTGGTGGGGTCATACAA   480
AdTDC10      CACATGTTGAAGCTTCCTAAATCTTTCATGTTTCAAGGCACTGGTGGTGGGGTCATACAA   480
AdTDC11      CACATGTTGAAGCTTCCTAAATCTTTCATGTTTCAAGGCACGGGTGGTGGGGTCATACAA   480
AdTDC12      CACATGTTGAAGCTTCCTAAATCTTTCATGTTTCAAGGCACGGGTGGTGGGGTCATACAA   480
AdTDC13      CACATGTTGAAGCTTCCTAAATCTTTCATGTTTCAAGGCACGGGTGGTGGGGTCATACAA   480
AdTDC14      CACATGTTGAAGCTTCCTAAATCTTTCATGTTTCAAGGCACGGGTGGTGGGGTCATACAA   480
```

```
AdTDC15      CACATGTTGAAGCTTCCTAAATCTTTCATGTTTCAAGGCACGGGTGGTGGGGTCATACAA    480
AdTDC16      CACATGTTGAAGCTTCCTAAATCTTTCATGTTTCAAGGCACGGGTGGTGGGGTCATACAA    480
AdTDC17      CACATGTTGAAGCTTCCTAAATCTTTCATGTTTCAAGGCACGGGTGGTGGGGTCATACAA    480
AdTDC18      CACATGTTGAAGCTTCCTAAATCTTTCATGTTTCAAGGCACGGGTGGTGGGGTCATACAA    480
AdTDC19      CACATGTTGAAGCTTCCTAAATCTTTCATGTTTCAAGGCACGGGTGGTGGGGTCATACAA    480
AdTDC20      CACATGTTGAAGCTTCCTAAATCTTTCATGTTTCAAGGCACGGGTGGTGGGGTCATACAA    480
             ***************************************** ******************

Achn173261   GGCACAACTAGTGACTCGATCCTCTGCACCCTCGTCGCCGCGAGAAACCGTGCACTCGAG    540
AdTDC1       GGCACAACTAGTGACTCGATCCTCTGCACCCTCGTTGCCGCGAGAGACCGTGCACTCGAG    540
AdTDC2       GGCACAACTAGTGACTCGATCCTCTGCACCCTCGTTGCCGCGAGAGACCGTGCACTCGAG    540
AdTDC3       GGCACAACTAGTGACTCGATCCTCTGCACCCTCGTTGCCGCGAGAGACCGTGCACTCGAG    540
AdTDC4       GGCACAACTAGTGACTCGATCCTCTGCACCCTCGTTGCCGCGAGAGACCGTGCACTCGAG    540
AdTDC5       GGCACAACTAGTGACTCGATCCTCTGCACCCTCGTTGCCGCGAGAGACCGTGCACTCGAG    540
AdTDC6       GGCACAACTAGTGACTCGATCCTCTGCACCCTCGTTGCCGCGAGAGACCGTGCACTCGAG    540
AdTDC7       GGCACAACTAGTGACTCGATCCTCTGCACCCTCGTTGCCGCGAGAGACCGTGCACTCGAG    540
AdTDC8       GGCACAACTAGTGACTCGATCCTCTGCACCCTCGTTGCCGCGAGAGACCGTGCACTCGAG    540
AdTDC9       GGCACAACTAGTGACTCGATCCTCTGCACCCTCGTTGCCGCGAGAGACCGTGCACTCGAG    540
AdTDC10      GGCACAACTAGTGACTCGATCCTCTGCACCCTCGTTGCCGCGAGAGACCGTGCACTCGAG    540
AdTDC11      GGCACAACTAGTGACTCGATCCTCTGCACCCTCGTCGCCGCGAGAAACCGTACACTCGAG    540
AdTDC12      GGCACAACTAGTGACTCGATCCTCTGCACCCTCGTCGCCGCGAGAAACCGTACACTCGAG    540
AdTDC13      GGCACAACTAGTGACTCGATCCTCTGCACCCTCGTCGCCGCGAGAAACCGTACACTCGAG    540
AdTDC14      GGCACAACTAGTGACTCGATCCTCTGCACCCTCGTCGCCGCGAGAAACCGTACACTCGAG    540
AdTDC15      GGCACAACTAGTGACTCGATCCTCTGCACCCTCGTCGCCGCGAGAAACCGTACACTCGAG    540
AdTDC16      GGCACAACTAGTGACTCGATCCTCTGCACCCTCGTCGCCGCGAGAAACCGTGCACTCGAG    540
AdTDC17      GGCACAACTAGTGACTCGATCCTCTGCACCCTCGTCGCCGCGAGAAACCGTGCACTCGAG    540
AdTDC18      GGCACAACTAGTGACTCGATCCTCTGCACCCTCGTCGCCGCGAGAAACCGTGCACTCGAG    540
AdTDC19      GGCACAACTAGTGACTCGATCCTCTGCACCCTCGTCGCCGCGAGAAACCGTGCACTCGAG    540
AdTDC20      GGCACAACTAGTGACTCGATCCTCTGCACCCTCGTCGCCGCGAGAAACCGTGCACTCGAG    540
             ********************************** ********** ***** ********

Achn173261   AACGTGGGTGTTGATAATATCGGTAAGCTCGTCGTGTACGGCTCTGATCAGACTCATTCT    600
AdTDC1       AACGTGGGCGTTGATAATATCGGTAAGCTCGTCGTGTATGGCTCTGATCAGACTCATTCT    600
AdTDC2       AACGTGGGCGTTGATAATATCGGTAAGCTCGTCGTGTATGGCTCTGATCAGACTCATTCT    600
AdTDC3       AACGTGGGCGTTGATAATATCGGTAAGCTCGTCGTGTATGGCTCTGATCAGACTCATTCT    600
AdTDC4       AACGTGGGCGTTGATAATATCGGTAAGCTCGTCGTGTATGGCTCTGATCAGACTCATTCT    600
AdTDC5       AACGTGGGCGTTGATAATATCGGTAAGCTCGTCGTGTATGGCTCTGATCAGACTCATTCT    600
AdTDC6       AACGTGGGCGTTGATAATATCGGTAAGCTCGTCGTGTATGGCTCTGATCAGACTCATTCT    600
AdTDC7       AACGTGGGCGTTGATAATATCGGTAAGCTCGTCGTGTATGGCTCTGATCAGACTCATTCT    600
AdTDC8       AACGTGGGCGTTGATAATATCGGTAAGCTCGTCGTGTATGGCTCTGATCAGACTCATTCT    600
AdTDC9       AACGTGGGCGTTGATAATATCGGTAAGCTCGTCGTGTATGGCTCTGATCAGACTCATTCT    600
AdTDC10      AACGTGGGCGTTGATAATATCGGTAAGCTCGTCGTGTATGGCTCTGATCAGACTCATTCT    600
AdTDC11      AACGTGGGCGTTGATAATATCGGTAAGCTCGTCGTGTACGGCTCTGATCAGACTCATTCT    600
AdTDC12      AACGTGGGCGTTGATAATATCGGTAAGCTCGTCGTGTACGGCTCTGATCAGACTCATTCT    600
```

```
AdTDC13      AACGTGGGCGTTGATAATATCGGTAAGCTCGTCGTGTACGGCTCTGATCAGACTCATTCT   600
AdTDC14      AACGTGGGCGTTGATAATATCGGTAAGCTCGTCGTGTACGGCTCTGATCAGACTCATTCT   600
AdTDC15      AACGTGGGCGTTGATAATATCGGTAAGCTCGTCGTGTACGGCTCTGATCAGACTCATTCT   600
AdTDC16      AACGTGGGCGTTGATAATATCGGTAAGCTCGTCGTGTACGGCTCTGATCAGACTCATTCT   600
AdTDC17      AACGTGGGCGTTGATAATATCGGTAAGCTCGTCGTGTACGGCTCTGATCAGACTCATTCT   600
AdTDC18      AACGTGGGCGTTGATAATATCGGTAAGCTCGTCGTGTACGGCTCTGATCAGACTCATTCT   600
AdTDC19      AACGTGGGCGTTGATAATATCGGTAAGCTCGTCGTGTACGGCTCTGATCAGACTCATTCT   600
AdTDC20      AACGTGGGCGTTGATAATATCGGTAAGCTCGTCGTGTACGGCTCTGATCAGACTCATTCT   600
             ******** ***************************** *********************

Achn173261   ACTTATACCAAGGCGTGTAAGCTGGCGGGGATATATCCGCGCAACATACGATCGGTGCCC   660
AdTDC1       ACTTACACCAAGGCGTGTAAGCTGGCGGGGATATATCCGCGCAACATACGATCGGTGCCC   660
AdTDC2       ACTTACACCAAGGCGTGTAAGCTGGCGGGGATATATCCGCGCAACATACGATCGGTGCCC   660
AdTDC3       ACTTACACCAAGGCGTGTAAGCTGGCGGGGATATATCCGCGCAACATACGATCGGTGCCC   660
AdTDC4       ACTTACACCAAGGCGTGTAAGCTGGCGGGGATATATCCGCGCAACATACGATCGGTGCCC   660
AdTDC5       ACTTACACCAAGGCGTGTAAGCTGGCGGGGATATATCCGCGCAACATACGATCGGTGCCC   660
AdTDC6       ACTTACACCAAGGCGTGTAAGCTGGCGGGGATATATCCGCGCAACATACGATCGGTGCCC   660
AdTDC7       ACTTACACCAAGGCGTGTAAGCTGGCGGGGATATATCCGCGCAACATACGATCGGTGCCC   660
AdTDC8       ACTTACACCAAGGCGTGTAAGCTGGCGGGGATATATCCGCGCAACATACGATCGGTGCCC   660
AdTDC9       ACTTACACCAAGGCGTGTAAGCTGGCGGGGATATATCCGCGCAACATACGATCGGTGCCC   660
AdTDC10      ACTTACACCAAGGCGTGTAAGCTGGCGGGGATATATCCGCGCAACATACGATCGGTGCCC   660
AdTDC11      ACTTATACCAAGGCGTGTAAGCTGGCGGGGATATATCCGCGCAACATACGATCGGTGCCC   660
AdTDC12      ACTTATACCAAGGCGTGTAAGCTGGCGGGGATATATCCGCGCAACATACGATCGGTGCCC   660
AdTDC13      ACTTATACCAAGGCGTGTAAGCTGGCGGGGATATATCCGCGCAACATACGATCGGTGCCC   660
AdTDC14      ACTTATACCAAGGCGTGTAAGCTGGCGGGGATATATCCGCGCAACATACGATCGGTGCCC   660
AdTDC15      ACTTATACCAAGGCGTGTAAGCTGGCGGGGATATATCCGCGCAACATACGATCGGTGCCC   660
AdTDC16      ACTTATACCAAGGCGTGTAAGCTGGCGGGGATATATCCGCGCAACATACGATCGGTGCCC   660
AdTDC17      ACTTATACCAAGGCGTGTAAGCTGGCGGGGATATATCCGCGCAACATACGATCGGTGCCC   660
AdTDC18      ACTTATACCAAGGCGTGTAAGCTGGCGGGGATATATCCGCGCAACATACGATCGGTGCCC   660
AdTDC19      ACTTATACCAAGGCGTGTAAGCTGGCGGGGATATATCCGCGCAACATACGATCGGTGCCC   660
AdTDC20      ACTTATACCAAGGCGTGTAAGCTGGCGGGGATATATCCGCGCAACATACGATCGGTGCCC   660
             ***** ******************************************************

Achn173261   GCAGGGTCCGACACGTGTTTCGCTATGTCCCCTGTGGCGCTGAGGAAGGTGATAGATGCT   720
AdTDC1       GCAGGGTCCAACACGTGTTTCGCTATGTCCCCTGTGGCGCTGAGGAAAGTGATAGATACT   720
AdTDC2       GCAGGGTCCAACACGTGTTTCGCTATGTCCCCTGTGGCGCTGAGGAAGGTGATAGATACT   720
AdTDC3       GCAGGGTCCAACACGTGTTTCGCTATGTCCCCTGTGGCGCTGAGGAAGGTGATAGATACT   720
AdTDC4       GCAGGGTCCAACACGTGTTTCGCTATGTCCCCTGTGGCGCTGAGGAAGGTGATAGATACT   720
AdTDC5       GCAGGGTCCAACACGTGTTTCGCTATGTCCCCTGTGGCGCTGAGGAAGGTGATAGATACT   720
AdTDC6       GCAGGGTCCAACACGTGTTTCGCTATGTCCCCTGTGGCGCTGAGGAAGGTGATAGATACT   720
AdTDC7       GCAGGGTCCAACACGTGTTTCGCTATGTCCCCTGTGGCGCTGAGGAAGGTGATAGATACT   720
AdTDC8       GCAGGGTCCAACACGTGTTTCGCTATGTCCCCTGTGGCGCTGAGGAAGGTGATAGATACT   720
AdTDC9       GCAGGGTCCGACACGTGTTTCGCTATGTCCCCTGTGGCGCTGAGGAAGGTGATAGATGCT   720
AdTDC10      GCAGGGTCCGACACGTGTTTCGCTATGTCCCCTGTGGCGCTGAGGAAGGTGATAGATGCT   720
```

```
AdTDC11      GCAGGGTCCGACACGTGTTTCGCTATGTCCCCTGTGGCGCTGAGGAAGGTGATAGATGCT   720
AdTDC12      GCAGGGTCCGACACGTGTTTCGCTATGTCCCCTGTGGCGCTGAGGAAGGTGATAGATGCT   720
AdTDC13      GCAGGGTCCGACACGTGTTTCGCTATGTCCCCTGTGGCGCTGAGGAAGGTGATAGATGCT   720
AdTDC14      GCAGGGTCCGACACGTGTTTCGCTATGTCCCCTGTGGCGCTGAGGAAGGTGATAGATGCT   720
AdTDC15      GCAGGGTCCGACACGTGTTTCGCTATGTCCCCTGTGGCGCTGAGGAAGGTGATAGATGCT   720
AdTDC16      GCAGGGTTCGACACGTGTTTCGCTATGTCCCCTGTGGCGCTGAGGAAAGTGATAGATGCT   720
AdTDC17      GCAGGGTTCGACACGTGTTTCGCTATGTCCCCTGTGGCGCTGAGGAAGGTGATAGATGCT   720
AdTDC18      GCAGGGTTCGACACGTGTTTCGCTATGTCCCCTGTGGCGCTGAGGAAGGTGATAGATGCT   720
AdTDC19      GCAGGGTTCGACACGTGTTTCGCTATGTCCCCTGTGGCGCTGAGGAAGGTGATAGATGCT   720
AdTDC20      GCAGGGTTCGACACGTGTTTCGCTATGTCCCCTGTGGCGCTGAGGAAGGTGATAGATGCT   720
             ******* * ************************************* ********* **

Achn173261   GACCTGGCAGCCGGGATGGTCCCACTTTATTTGTGTGTGACTGTGGGGACAACTTCAACC   780
AdTDC1       GACGTGGCAGCCGGGCTGGTCCCACTTTATTTGTGTGTGACTGTGGGGACAACTTCAACC   780
AdTDC2       GACGTGGCAGCCGGGCTGGTCCCACTTTATTTGTGTGTGACTGTGGGGACAACTTCAACC   780
AdTDC3       GACGTGGCAGCCGGGCTGGTCCCACTTTATTTGTGTGTGACTGTGGGGACAACTTCAACC   780
AdTDC4       GACGTGGCAGCCGGGCTGGTCCCACTTTATTTGTGTGTGACTGTGGGGACAACTTCAACC   780
AdTDC5       GACGTGGCAGCCGGGCTGGTCCCACTTTATTTGTGTGTGACTGTGGGGACAACTTCAACC   780
AdTDC6       GACGTGGCAGCCGGGCTGGTCCCACTTTATTTGTGTGTGACTGTGGGGACAACTTCAACC   780
AdTDC7       GACGTGGCAGCCGGGCTGGTCCCACTTTATTTGTGTGTGACTGTGGGGACAACTTCAACC   780
AdTDC8       GACGTGGCAGCCGGGCTGGTCCCACTTTATTTGTGTGTGACTGTGGGGACAACTTCAACC   780
AdTDC9       GACGTGGCAGCCGGGCTGGTCCCACTTTATTTGTGTGTGACTGTGGGGACAACTTCAACG   780
AdTDC10      GACGTGGCAGCCGGGCTGGTCCCACTTTATTTGTGTGTGACTGTGGGGACAACTTCAACG   780
AdTDC11      GACCTGGCAGCCGGGTTGGTCCCACTTTATTTGTGTGTGACTGTGGGGACAACTTCAACC   780
AdTDC12      GACCTGGCAGCCGGGTTGGTCCCACTTTATTTGTGTGTGACTGTGGGGACAACTTCAACC   780
AdTDC13      GACCTGGCAGCCGGGTTGGTCCCACTTTATTTGTGTGTGACTGTGGGGACAACTTCAACC   780
AdTDC14      GACCTGGCAGCCGGGTTGGTCCCACTTTATTTGTGTGTGACTGTGGGGACAACTTCAACC   780
AdTDC15      GACCTGGCAGCCGGGTTGGTCCCACTTTATTTGTGTGTGACTGTGGGGACAACTTCAACC   780
AdTDC16      GACCTGGCAGCCGGGCTGGTCCCACTTTATTTGTGTGTGACTGTGGGGACAACTTCAACC   780
AdTDC17      GACCTGGCAGCCGGGCTGGTCCCACTTTATTTGTGTGTGACTGTGGGGACAACTTCAACC   780
AdTDC18      GACCTGGCAGCCGGGCTGGTCCCACTTTATTTGTGTGTGACTGTGGGGACAACTTCAACC   780
AdTDC19      GACCTGGCAGCCGGGCTGGTCCCACTTTATTTGTGTGTGACTGTGGGGACAACTTCAACC   780
AdTDC20      GACCTGGCAGCCGGGTTGGTCCCACTTTATTTGTGTGTGACTGTGGGGACAACTTCAACC   780
             *** *********** *******************************************

Achn173261   ACTGCGGTTGATCCGCTGGAGGACCTTGCTGATGTTGCGAACGATTATGGCGTTTGGGTC   840
AdTDC1       ACTCCGGTTGATCCGCTGGAGGACCTTGCTGATGTTGCGAACGATTACGGCGTTTGGATC   840
AdTDC2       ACTCCGGTTGATCCGCTGGAGGACCTTGCTGATGTTGCGAACGATTACGGCGTTTGGATC   840
AdTDC3       ACTCCGGTTGATCCGCTGGAGGACCTTGCTGATGTTGCGAACGATTACGGCGTTTGGATC   840
AdTDC4       ACTCCGGTTGATCCGCTGGAGGACCTTGCTGATGTTGCGAACGATTACGGCGTTTGGATC   840
AdTDC5       ACTCCGGTTGATCCGCTGGAGGACCTTGCTGATGTTGCGAACGATTACGGCGTTTGGATC   840
AdTDC6       ACTCCGGTTGATCCGCTGGAGGACCTTGCTGATGTTGCGAACGATTACGGCGTTTGGATC   840
AdTDC7       ACTCCGGTTGATCCGCTGGAGGACCTTGCTGATGTTGCGAACGATTACGGCGTTTGGATC   840
AdTDC8       ACTCCGGTTGATCCGCTGGAGGACCTTGCTGATGTTGCGAACGATTACGGCGTTTGGATC   840
```

AdTDC9   ACTGCGGTTGATCCGCTGGAGGACCTTGCTGATGTTGCTAACGATTACGGCGTTTGGGTC   840
AdTDC10   ACTGCGGTTGATCCGCTGGAGGACCTTGCTGATGTTGCTAACGATTACGGCGTTTGGGTC   840
AdTDC11   ACTGCGGTTGATCCGCTGGAGGACCTTGCTGATGTTGCGAACGATTATGGCGTTTGGGTC   840
AdTDC12   ACTGCGGTTGATCCGCTGGAGGACCTTGCTGATGTTGCGAACGATTATGGCGTTTGGGTC   840
AdTDC13   ACTGCGGTTGATCCGCTGGAGGACCTTGCTGATGTTGCGAACGATTATGGCGTTTGGGTC   840
AdTDC14   ACTGCGGTTGATCCGCTGGAGGACCTTGCTGATGTTGCGAACGATTATGGCGTTTGGGTC   840
AdTDC15   ACTGCGGTTGATCCGCTGGAGGACCTTGCTGATGTTGCGAACGATTATGGCGTTTGGGTC   840
AdTDC16   ACTGCGGTTGATCCGCTGGAGGACCTTGCTGATGTTGCGACCGATTATGGCGTTTGGGTC   840
AdTDC17   ACTGCGGTTGATCCGCTGGAGGACCTTGCTGATGTTGCGACCGATTATGGCGTTTGGGTC   840
AdTDC18   ACTGCGGTTGATCCGCTGGAGGACCTTGCTGATGTTGCGACCGATTATGGCGTTTGGGTC   840
AdTDC19   ACTGCGGTTGATCCGCTGGAGGACCTTGCTGATGTTGCGACCGATTATGGCGTTTGGGTC   840
AdTDC20   ACTGCGGTTGATCCGCTGGAGGACCTTGCTGATGTTGCGAACGATTATGGCGTTTGGGTC   840
            *** ********************************** * ****** ********* **

Achn173261 CACGTGGACGCTGCCTACGGAGGGAGCGCGTGTATTTGCCCAGAGTTCCGGCACTACCTG   900
AdTDC1   CACGTGGACGCTGCCTACGGAGGGAGCGCGTGTATTTGCCCAGAGTTCCGGCACTACCTG   900
AdTDC2   CACGTGGACGCTGCCTACGGAGGGAGCGCGTGTATTTGCCCAGAGTTCCGGCACTACCTG   900
AdTDC3   CACGTGGACGCTGCCTACGGAGGGAGCGCGTGTATTTGCCCAGAGTTCCGGCACTACCTG   900
AdTDC4   CACGTGGACGCTGCCTACGGAGGGAGCGCGTGTATTTGCCCAGAGTTCCGGCACTACCTG   900
AdTDC5   CACGTGGACGCTGCCTACGGAGGGAGCGCGTGTATTTGCCCAGAGTTCCGGCACTACCTG   900
AdTDC6   CACGTGGACGCTGCCTACGGAGGGAGCGCGTGTATTTGCCCAGAGTTCCGGCACTACCTG   900
AdTDC7   CACGTGGACGCTGCCTACGGAGGGAGCGCGTGTATTTGCCCAGAGTTCCGGCACTACCTG   900
AdTDC8   CACGTGGACGCTGCCTACGGAGGGAGCGCGTGTATTTGCCCAGAGTTCCGGCACTACCTG   900
AdTDC9   CACGTGGACGCTGCCTACGGAGGGAGCGCGTGTATTTGCCCAGAGTTCCGGCACTACCTG   900
AdTDC10   CACGTGGACGCTGCCTACGGAGGGAGCGCGTGTATTTGCCCAGAGTTCCGGCACTACCTG   900
AdTDC11   CACGTGGACGCTGCCTACGGAGGGAGCGCGTGTATTTGCCCAGAGTTCCGGCACTACCTG   900
AdTDC12   CACGTGGACGCTGCCTACGGAGGGAGCGCGTGTATTTGCCCAGAGTTCCGGCACTACCTG   900
AdTDC13   CACGTGGACGCTGCCTACGGAGGGAGCGCGTGTATTTGCCCAGAGTTCCGGCACTACCTG   900
AdTDC14   CACGTGGACGCTGCCTACGGAGGGAGCGCGTGTATTTGCCCAGAGTTCCGGCACTACCTG   900
AdTDC15   CACGTGGACGCTGCCTACGGAGGGAGCGCGTGTATTTGCCCAGAGTTCCGGCACTACCTG   900
AdTDC16   CACGTGGACGCTGCCTACGGAGGGAGCGCGTGTATTTGCCCAGAGTTCCGGCACTACCTG   900
AdTDC17   CACGTGGACGCTGCCTACGGAGGGAGCGCGTGTATTTGCCCAGAGTTCCGGCACTACCTG   900
AdTDC18   CACGTGGACGCTGCCTACGGAGGGAGCGCGTGTATTTGCCCAGAGTTCCGGCACTACCTG   900
AdTDC19   CACGTGGACGCTGCCTACGGAGGGAGCGCGTGTATTTGCCCAGAGTTCCGGCACTACCTG   900
AdTDC20   CACGTGGACGCTGCCTACGGAGGGAGCGCGTGTATTTGCCCAGAGTTCCGGCACTACCTG   900
            ****

Achn173261 GACGGGATCGAGCTAGTCGACTCACTGAGTCTGAGCCCGCACAAGTGGCTGCTCAGTTAC   960
AdTDC1   GACGGGATCGAGCTAGTTGACTCACTGAGTCTGAGCCCGCACAAGTGGCTGCTCAGTTAC   960
AdTDC2   GACGGGATCGAGCTAGTTGACTCACTGAGTCTGAGCCCGCACAAGTGGCTGCTCAGTTAC   960
AdTDC3   GACGGGATCGAGCTAGTTGACTCACTGAGTCTGAGCCCGCACAAGTGGCTGCTCAGTTAC   960
AdTDC4   GACGGGATCGAGCTAGTTGACTCACTGAGTCTGAGCCCGCACAAGTGGCTGCTCAGTTAC   960
AdTDC5   GACGGGATCGAGCTAGTTGACTCACTGAGTCTGAGCCCGCACAAGTGGCTGCTCAGTTAC   960
AdTDC6   GACGGGATCGAGCTAGTTGACTCACTGAGTCTGAGCCCGCACAAGTGGCTGCTCAGTTAC   960

```
AdTDC7       GACGGGATCGAGCTAGTTGACTCACTGAGTCTGAGCCCGCACAAGTGGCTGCTCAGTTAC    960
AdTDC8       GACGGGATCGAGCTAGTTGACTCACTGAGTCTGAGCCCGCACAAGTGGCTGCTCAGTTAC    960
AdTDC9       GACGGGATCGAGCAAGTCGACTCACTGAGTCTGAGCCCGCACAAGTGGCTGCTCAGTTAC    960
AdTDC10      GACGGGATCGAGCAAGTCGACTCACTGAGTCTGAGCCCGCACAAGTGGCTGCTCAGTTAC    960
AdTDC11      GACGGGATCGAGCTAGTCGACTCACTGAGTCTGAGCCCGCACAAGTGGCTGCTCAGTTAC    960
AdTDC12      GACGGGATCGAGCTAGTCGACTCACTGAGTCTGAGCCCGCACAAGTGGCTGCTCAGTTAC    960
AdTDC13      GACGGGATCGAGCTAGTCGACTCACTGAGTCTGAGCCCGCACAAGTGGCTGCTCAGTTAC    960
AdTDC14      GACGGGATCGAGCTAGTCGACTCACTGAGTCTGAGCCCGCACAAGTGGCTGCTCAGTTAC    960
AdTDC15      GACGGGATCGAGCTAGTCGACTCACTGAGTCTGAGCCCGCACAAGTGGCTGCTCAGTTAC    960
AdTDC16      GACGGGATCGAGCTAGTCGACTCACTGAGTCTGAGCCCGCACAAGTGGCTGCTCAGTTAC    960
AdTDC17      GACGGGATCGAGCTAGTCGACTCACTGAGTCTGAGCCCGCACAAGTGGCTGCTCAGTTAC    960
AdTDC18      GACGGGATCGAGCTAGTCGACTCACTGAGTCTGAGCCCGCACAAGTGGCTGCTCAGTTAC    960
AdTDC19      GACGGGATCGAGCTAGTCGACTCACTGAGTCTGAGCCCGCACAAGTGGCTGCTCAGTTAC    960
AdTDC20      GACGGGATCGAGCTAGTCGACTCACTGAGTCTGAGCCCGCACAAGTGGCTGCTCAGTTAC    960
             ************* *** ******************************************

Achn173261   TTGGACTGCTGTTGCTTGTGGGTGAAGCAACCCGGATTGCTAGTCAAAGCCTTGAGCACT    1020
AdTDC1       TTGGACTGCTGTTGCTTGTGGGTGAAGAAACCCGGATTGCTAGTCAAAGCCTTGAGCACT    1020
AdTDC2       TTGGACTGCTGTTGCTTGTGGGTGAAGAAACCCGGATTGCTAGTCAAAGCCTTGAGCACT    1020
AdTDC3       TTGGACTGCTGTTGCTTGTGGGTGAAGAAACCCGGATTGCTAGTCAAAGCCTTGAGCACT    1020
AdTDC4       TTGGACTGCTGTTGCTTGTGGGTGAAGAAACCCGGATTGCTAGTCAAAGCCTTGAGCACT    1020
AdTDC5       TTGGACTGCTGTTGCTTGTGGGTGAAGAAACCCGGATTGCTAGTCAAAGCCTTGAGCACT    1020
AdTDC6       TTGGACTGCTGTTGCTTGTGGGTGAAGAAACCCGGATTGCTAGTCAAAGCCTTGAGCACT    1020
AdTDC7       TTGGACTGCTGTTGCTTGTGGGTGAAGAAACCCGGATTGCTAGTCAAAGCCTTGAGCACT    1020
AdTDC8       TTGGACTGCTGTTGCTTGTGGGTGAAGAAACCCGGATTGCTAGTCAAAGCCTTGAGCACT    1020
AdTDC9       TTGGACTGCTGTTGCTTGTGGGTGAAGAAACCCGGATTGCTAGTCAAAGCCTTGAGCACT    1020
AdTDC10      TTGGACTGCTGTTGCTTGTGGGTGAAGAAACCCGGATTGCTAGTCAAAGCCTTGAGCACT    1020
AdTDC11      TTGGACTGCTGTTGCTTGTGGGTGAAGCAACCCGGATTGCTAGTCAAAGCCTTGAGCACT    1020
AdTDC12      TTGGACTGCTGTTGCTTGTGGGTGAAGCAACCCGGATTGCTAGTCAAAGCCTTGAGCACT    1020
AdTDC13      TTGGACTGCTGTTGCTTGTGGGTGAAGCAACCCGGATTGCTAGTCAAAGCCTTGAGCACT    1020
AdTDC14      TTGGACTGCTGTTGCTTGTGGGTGAAGCAACCCGGATTGCTAGTCAAAGCCTTGAGCACT    1020
AdTDC15      TTGGACTGCTGTTGCTTGTGGGTGAAGCAACCCGGATTGCTAGTCAAAGCCTTGAGCACT    1020
AdTDC16      TTGGACTGCTGTTGCTTGTGGGTGAAGCAACCCGGATTGCTAGTCAAAGCCTTGAGCACT    1020
AdTDC17      TTGGACTGCTGTTGCTTGTGGGTGAAGCAACCCGGATTGCTAGTCAAAGCCTTGAGCACT    1020
AdTDC18      TTGGACTGCTGTTGCTTGTGGGTGAAGCAACCCGGATTGCTAGTCAAAGCCTTGAGCACT    1020
AdTDC19      TTGGACTGCTGTTGCTTGTGGGTGAAGCAACCCGGATTGCTAGTCAAAGCCTTGAGCACT    1020
AdTDC20      TTGGACTGCTGTTGCTTGTGGGTGAAGCAACCCGGATTGCTAGTCAAAGCCTTGAGCACT    1020
             *************************** ********************************

Achn173261   AACCCGGAGTACTTGAGAAACAAACCCAGAGAGTCGGACTCGGTGGTGGACTACAAGGAC    1080
AdTDC1       AACCCGGAGTACTTGAGAAACAAACCCAGTGAGTCGGACTCGGTGGTGGACTACAAGGAC    1080
AdTDC2       AACCCGGAGTACTTGAGAAACAAACCCAGTGAGTCGGACTCGGTGGTGGACTACAAGGAC    1080
AdTDC3       AACCCGGAGTACTTGAGAAACAAACCCAGTGAGTCGGACTCGGTGGTGGACTACAAGGAC    1080
AdTDC4       AACCCGGAGTACTTGAGAAACAAACCCAGTGAGTCGGACTCGGTGGTGGACTACAAGGAC    1080
```

```
AdTDC5       AACCCGGAGTACTTGAGAAACAAACCCAGTGAGTCGGACTCGGTGGTGGACTACAAGGAC    1080
AdTDC6       AACCCGGAGTACTTGAGAAACAAACCCAGTGAGTCGGACTCGGTGGTGGACTACAAGGAC    1080
AdTDC7       AACCCGGAGTACTTGAGAAACAAACCCAGTGAGTCGGACTCGGTGGTGGACTACAAGGAC    1080
AdTDC8       AACCCGGAGTACTTGAGAAACAAACCCAGTGAGTCGGACTCGGTGGTGGACTACAAGGAC    1080
AdTDC9       AACCCGGAGTACTTGAGAAACAAACCCAGTGAGTCGGACTCGGTGGTGGACTACAAGGAC    1080
AdTDC10      AACCCGGAGTACTTGAGAAACAAACCCAGTGAGTCGGACTCGGTGGTGGACTACAAGGAC    1080
AdTDC11      AACCCGGAGTACTTGAGAAACAAACCCAGTGAGTCGGACTCGGTGGTGGACTACAAGGAC    1080
AdTDC12      AACCCGGAGTACTTGAGAAACAAACCCAGTGAGTCGGACTCGGTGGTGGACTACAAGGAC    1080
AdTDC13      AACCCGGAGTACTTGAGAAACAAACCCAGTGAGTCGGACTCGGTGGTGGACTACAAGGAC    1080
AdTDC14      AACCCGGAGTACTTGAGAAACAAACCCAGTGAGTCGGACTCGGTGGTGGACTACAAGGAC    1080
AdTDC15      AACCCGGAGTACTTGAGAAACAAACCCAGTGAGTCGGACTCGGTGGTGGACTACAAGGAC    1080
AdTDC16      AACCCGGAGTACTTGAGAAACAAACCCAGTGAGTCGGATTCGGTGGTGGACTACAAGGAC    1080
AdTDC17      AACCCGGAGTACTTGAGAAACAAACCCAGTGAGTCGGATTCGGTGGTGGACTACAAGGAC    1080
AdTDC18      AACCCGGAGTACTTGAGAAACAAACCCAGTGAGTCGGATTCGGTGGTGGACTACAAGGAC    1080
AdTDC19      AACCCGGAGTACTTGAGAAACAAACCCAGTGAGTCGGATTCGGTGGTGGACTACAAGGAC    1080
AdTDC20      AACCCGGAGTACTTGAGAAACAAACCCAGTGAGTCGGACTCGGTGGTGGACTACAAGGAC    1080
             ***************************** ******** *********************

Achn173261   TGGCAAGTGGGTACGGGTCGTCGGTTCAAAGCGCTCCGACTCTGGCTCGTTTTACGTAGT    1140
AdTDC1       TGGCAAGTGGGTACGGGTCGTCGGTTCAAAGCGCTCCGACTCTGGCTCGTTTTACGTAGC    1140
AdTDC2       TGGCAAGTGGGTACGGGTCGTCGGTTCAAAGCGCTCCGACTCTGGCTCGTTTTACGTAGC    1140
AdTDC3       TGGCAAGTGGGTACGGGTCGTCGGTTCAAAGCGCTCCGACTCTGGCTCGTTTTACGTAGC    1140
AdTDC4       TGGCAAGTGGGTACGGGTCGTCGGTTCAAAGCGCTCCGACTCTGGCTCGTTTTACGTAGC    1140
AdTDC5       TGGCAAGTGGGTACGGGTCGTCGGTTCAAAGCGCTCCGACTCTGGCTCGTTTTACGTAGC    1140
AdTDC6       TGGCAAGTGGGTACGGGTCGTCGGTTCAAAGCGCTCCGACTCTGGCTCGTTTTACGTAGC    1140
AdTDC7       TGGCAAGTGGGTACGGGTCGTCGGTTCAAAGCGCTCCGACTCTGGCTCGTTTTACGTAGC    1140
AdTDC8       TGGCAAGTGGGTACGGGTCGTCGGTTCAAAGCGCTCCGACTCTGGCTCGTTTTACGTAGC    1140
AdTDC9       TGGCAAGTGGGTACGGGTCGTCGGTTCAAAGCGCTCCGACTCTGGCTCGTTTTACGTAGC    1140
AdTDC10      TGGCAAGTGGGTACGGGTCGTCGGTTCAAAGCGCTCCGACTCTGGCTCGTTTTACGTAGC    1140
AdTDC11      TGGCAAGTGGGTACGGGTCGTCGGTTCAAAGCGCTCCGACTCTGGCTCGTTTTACGTAGT    1140
AdTDC12      TGGCAAGTGGGTACGGGTCGTCGGTTCAAAGCGCTCCGACTCTGGCTCGTTTTACGTAGT    1140
AdTDC13      TGGCAAGTGGGTACGGGTCGTCGGTTCAAAGCGCTCCGACTCTGGCTCGTTTTACGTAGT    1140
AdTDC14      TGGCAAGTGGGTACGGGTCGTCGGTTCAAAGCGCTCCGACTCTGGCTCGTTTTACGTAGT    1140
AdTDC15      TGGCAAGTGGGTACGGGTCGTCGGTTCAAAGCGCTCCGACTCTGGCTCGTTTTACGTAGT    1140
AdTDC16      TGGCAAGTGGGTACGGGTCGTCGGTTCAAAGCGCTCCGACTCTGGCTCGTTTTACGTAGC    1140
AdTDC17      TGGCAAGTGGGTACGGGTCGTCGGTTCAAAGCGCTCCGACTCTGGCTCGTTTTACGTAGC    1140
AdTDC18      TGGCAAGTGGGTACGGGTCGTCGGTTCAAAGCGCTCCGACTCTGGCTCGTTTTACGTAGC    1140
AdTDC19      TGGCAAGTGGGTACGGGTCGTCGGTTCAAAGCGCTCCGACTCTGGCTCGTTTTACGTAGC    1140
AdTDC20      TGGCAAGTGGGTACGGGTCGTCGGTTCAAAGCGCTCCGACTCTGGCTCGTTTTACGTAGT    1140
             *********************************************************** 

Achn173261   TATGGGATCGCCAACCTCCAGAGCCACATCCGGTCCGATGTCAGGATGGCCAAGATGTTC    1200
AdTDC1       TATGGGATCGCCAACCTCCAGAGCCACATCCGGTCCGACGTCAGGATGGCCAAGATGTTC    1200
AdTDC2       TATGGGATCGCCAACCTCCAGAGCCACATCCGGTCCGACGTCAGGATGGCCAAGATGTTC    1200
```

```
AdTDC3      TATGGGATCGCCAACCTCCAGAGCCACATCCGGTCCGACGTCAGGATGGCCAAGATGTTC   1200
AdTDC4      TATGGGATCGCCAACCTCCAGAGCCACATCCGGTCCGACGTCAGGATGGCCAAGATGTTC   1200
AdTDC5      TATGGGATCGCCAACCTCCAGAGCCACATCCGGTCCGACGTCAGGATGGCCAAGATGTTC   1200
AdTDC6      TATGGGATCGCCAACCTCCAGAGCCACATCCGGTCCGACGTCAGGATGGCCAAGATGTTC   1200
AdTDC7      TATGGGATCGCCAACCTCCAGAGCCACATCCGGTCCGACGTCAGGATGGCCAAGATGTTC   1200
AdTDC8      TATGGGATCGCCAACCTCCAGAGCCACATCCGGTCCGACGTCAGGATGGCCAAGATGTTC   1200
AdTDC9      TATGGGATCGCCAACCTCCAGAGCCACATCCGGTCCGACGTCAGGATGGCCAAGATGTTC   1200
AdTDC10     TATGGGATCGCCAACCTCCAGAGCCACATCCGGTCCGACGTCAGGATGGCCAAGATGTTC   1200
AdTDC11     TATGGGATCGCCAACCTCCAGAGCCACATCCGGTCCGATGTCAGGATGGCCAAGATGTTC   1200
AdTDC12     TATGGGATCGCCAACCTCCAGAGCCACATCCGGTCCGATGTCAGGATGGCCAAGATGTTC   1200
AdTDC13     TATGGTATCGCCAACCTCCAGAGCCACATCCGGTCCGATGTCAGGATGGCCAAGATGTTC   1200
AdTDC14     TATGGGATCGCCAACCTCCAGAGCCACATCCGGTCCGATGTCAGGATGGCCAAGATGTTC   1200
AdTDC15     TATGGGATCGCCAACCTCCAGAGCCACATCCGGTCCGATGTCAGGATGGCCAAGATGTTC   1200
AdTDC16     TATGGGATCGCCAACCTCCAGAGCCACATCCGGTCCGACGTCAGGATGGCCAAGATGTTC   1200
AdTDC17     TATGGGATCGCCAACCTCCAGAGCCACATCCGGTCCGACGTCAGGATGGCCAAGATGTTC   1200
AdTDC18     TATGGGATCGCCAACCTCCAGAGCCACATCCGGTCCGACGTCAGGATGGCCAAGATGTTC   1200
AdTDC19     TATGGGATCGCCAACCTCCAGAGCCACATCCGGTCCGACGTCAGGATGGCCAAGATGTTC   1200
AdTDC20     TATGGTATCGCCAACCTCCAGAGCCACATCCGGTCCGATGTCAGGATGGCCAAGATGTTC   1200
            ***** ******************************** *********************

Achn173261  GAGGGATTCGTGAAGTCCGACCCGCGATTCGAGTTGGTTGTGCCCCGGATCTTCTCACTG   1260
AdTDC1      GAGGGGTTCGTGAAGTCCGACCCGCGATTCGAGTTGGTCGTGCCCCGGATCTTCTCATTG   1260
AdTDC2      GAGGGGTTCGTGAAGTCCGACCCGCGATTCGAGTTGGTCGTGCCCCGGATCTTCTCATTG   1260
AdTDC3      GAGGGGTTCGTGAAGTCCGACCCGCGATTCGAGTTGGTCGTGCCCCGGATCTTCTCATTG   1260
AdTDC4      GAGGGGTTCGTGAAGTCCGACCCGCGATTCGAGTTGGTCGTGCCCCGGATCTTCTCATTG   1260
AdTDC5      GAGGGGTTCGTGAAGTCCGACCCGCGATTCGAGTTGGTCGTGCCCCGGATCTTCTCATTG   1260
AdTDC6      GAGGGGTTCGTGAAGTCCGACCCGCGATTCGAGTTGGTCGTGCCCCGGATCTTCTCATTG   1260
AdTDC7      GAGGGGTTCGTGAAGTCCGACCCGCGATTCGAGTTGGTCGTGCCCCGGATCTTCTCATTG   1260
AdTDC8      GAGGGGTTCGTGAAGTCCGACCCGCGATTCGAGTTGGTCGTGCCCCGGATCTTCTCATTG   1260
AdTDC9      GAGGGGTTCGTGAAGTCCGACCTGCGATTCGAGTTGGTCGTGCCCCGGATCTTCTCACTG   1260
AdTDC10     GAGGGGTTCGTGAAGTCCGACCTGCGATTCGAGTTGGTCGTGCCCCGGATCTTCTCACTG   1260
AdTDC11     GAGGGGTTCGTGAAGTCCGACCCGCGATTCGAGTTGGTTGTGCCCCGAATCTTCTCACTG   1260
AdTDC12     GAGGGGTTCGTGAAGTCCGACCCGCGATTCGAGTTGGTTGTGCCCCGAATCTTCTCACTG   1260
AdTDC13     GAGGGGTTCGTGAAGTCCGACCCGCGATTCGAGTTGGTTGTGCCCCGAATCTTCTCACTG   1260
AdTDC14     GAGGGGTTCGTGAAGTCCGACCCGCGATTCGAGTTGGTTGTGCCCCGAATCTTCTCACTG   1260
AdTDC15     GAGGGGTTCGTGAAGTCCGACCCGCGATTCGAGTTGGTTGTGCCCCGAATCTTCTCACTG   1260
AdTDC16     GAGGGGTTCGTGAAGTCCGACCTGCGATTCGAGTTGGTCGTGCCCCGGATCTTCTCATTG   1260
AdTDC17     GAGGGGTTCGTGAAGTCCGACCTGCGATTCGAGTTGGTCGTGCCCCGGATCTTCTCATTG   1260
AdTDC18     GAGGGGTTCGTGAAGTCCGACCTGCGATTCGAGTTGGTCGTGCCCCGGATCTTCTCATTG   1260
AdTDC19     GAGGGGTTCGTGAAGTCCGACCTGCGATTCGAGTTGGTCGTGCCCCGGATCTTCTCATTG   1260
AdTDC20     GAGGGGTTCGTGAAGTCCGACCCGCGATTCGAGTTGGTTGTGCCCCGAATCTTCTCACTG   1260
            ***** **************** *************** ******** ********* **

Achn173261  GTTTGCTTTCGTTTAAACCCGCACCCGGGATCATACCCGGGTTACCTGGAGCTTTTAAAC   1320
```

```
AdTDC1       GTTTGCTTCCGGTTAAACCCGCACCCGGGATCATACCCGGATTACCTGGAGCTTCTAAAC   1320
AdTDC2       GTTTGCTTCCGGTTAAACCCGCACCCGGGATCATACCCGGATTACCTGGAGCTTCTAAAC   1320
AdTDC3       GTTTGCTTCCGGTTAAACCCGCACCCGGGATCATACCCGGATTACCTGGAGCTTCTAAAC   1320
AdTDC4       GTTTGCTTCCGGTTAAACCCGCACCCGGGATCATACCCGGATTACCTGGAGCTTCTAAAC   1320
AdTDC5       GTTTGCTTCCGGTTAAACCCGCACCCGGGATCATACCCGGATTACCTGGAGCTTCTAAAC   1320
AdTDC6       GTTTGCTTCCGGTTAAACCCGCACCCGGGATCATACCCGGATTACCTGGAGCTTCTAAAC   1320
AdTDC7       GTTTGCTTCCGGTTAAACCCGCACCCGGGATCATACCCGGATTACCTGGAGCTTCTAAAC   1320
AdTDC8       GTTTGCTTCCGGTTAAACCCGCACCCGGGATCATACCCGGATTACCTGGAGCTTCTAAAC   1320
AdTDC9       GTTTGCTTCCGGTTAAACCCGCACCCGGGATCATACCCGGGTTACCTGGAGCTTTTAAAC   1320
AdTDC10      GTTTGCTTCCGGTTAAACCCGCACCCGGGATCATACCCGGGTTACCTGGAGCTTTTAAAC   1320
AdTDC11      GTTTGCTTTCGTTTAAACCCGCACCCGGGATCATACCCGGGTTACCTGGAGCTTTTAAAC   1320
AdTDC12      GTTTGCTTTCGTTTAAACCCGCACCCGGGATCATACCCGGGTTACCTGGAGCTTTTAAAC   1320
AdTDC13      GTTTGCTTTCGTTTAAACCCGCACCCGGGATCATACCCGGGTTACCTGGAGCTTTTAAAC   1320
AdTDC14      GTTTGCTTTCGTTTAAACCCGCACCCGGGATCATACCCGGGTTACCTGGAGCTTTTAAAC   1320
AdTDC15      GTTTGCTTTCGTTTAAACCCGCACCCGGGATCATACCCGGGTTACCTGGAGCTTTTAAAC   1320
AdTDC16      GTTTGCTTCCGGTTAAACCCGCACCCGGGATCATACCCGGATTATCTGGAGCTTCTAAAC   1320
AdTDC17      GTTTGCTTCCGGTTAAACCCGCACCCGGGATCATACCCGGATTATCTGGAGCTTCTAAAC   1320
AdTDC18      GTTTGCTTCCGGTTAAACCCGCACCCGGGATCATACCCGGATTATCTGGAGCTTCTAAAC   1320
AdTDC19      GTTTGCTTCCGGTTAAACCCGCACCCGGGATCATACCCGGATTATCTGGAGCTTCTAAAC   1320
AdTDC20      GTTTGCTTTCGTTTAAACCCGCACCCGGGATCATACCCGGATTATCTGGAGCTTCTAAAC   1320
             ******** ** **************************** *** ********* *****

Achn173261   AAAAAGCTACTTGAGTGGGTCAACTCAACGGGTGGACTCTACATGACCCACACAGTAGCG   1380
AdTDC1       AAAAAGCTCCTTGAGTGGGTCAACTCAACGGGTGGACTCTACATGACCCACACAGTAGCG   1380
AdTDC2       AAAAAGCTCCTTGAGTGGGTCAACTCAACGGGTGGACTCTACATGACCCACACAGTAGCG   1380
AdTDC3       AAAAAGCTCCTTGAGTGGGTCAACTCAACGGGTGGACTCTACATGACCCACACAGTAGCG   1380
AdTDC4       AAAAAGCTCCTTGAGTGGGTCAACTCAACGGGTGGACTCTACATGACCCACACAGTAGCG   1380
AdTDC5       AAAAAGCTCCTTGAGTGGGTCAACTCAACGGGTGGACTCTACATGACCCACACAGTAGCG   1380
AdTDC6       AAAAAGCTCCTTGAGTGGGTCAACTCAACGGGTGGACTCTACATGACCCACACAGTAGCG   1380
AdTDC7       AAAAAGCTCCTTGAGTGGGTCAACTCAACGGGTGGACTCTACATGACCCACACAGTAGCG   1380
AdTDC8       AAAAAGCTCCTTGAGTGGGTCAACTCAACGGGTGGACTCTACATGACCCACACAGTAGCG   1380
AdTDC9       AAAAAGCTCCTTGAGTGGGTCAACTCAACGGGTGGACTCTACATGACCCACACAGTAGCG   1380
AdTDC10      AAAAAGCTCCTTGAGTGGGTCAACTCAACGGGTGGACTCTACATGACCCACACAGTAGCG   1380
AdTDC11      AAAAAGCTCCTTGAGTGGGTCAACTCAACGGGTGGACTCTACATGACCCACACAGTAGCG   1380
AdTDC12      AAAAAGCTCCTTGAGTGGGTCAACTCAACGGGTGGACTCTACATGACCCACACAGTAGCG   1380
AdTDC13      AAAAAGCTCCTTGAGTGGGTCAACTCAACGGGTGGACTCTACATGACCCACACAGTAGCG   1380
AdTDC14      AAAAAGCTCCTTGAGTGGGTCAACTCAACGGGTGGACTCTACATGACCCACACAGTAGCG   1380
AdTDC15      AAAAAGCTCCTTGAGTGGGTCAACTCAACGGGTGGACTCTACATGACCCACACAGTAGCG   1380
AdTDC16      AAAAAGCTCCTTGAGTGGGTCAACTCAACGGGTGGACTCTACATGACCCACACAGTAGCG   1380
AdTDC17      AAAAAGCTCCTTGAGTGGGTCAACTCAACGGGTGGACTCTACATGACCCACACAGTAGCG   1380
AdTDC18      AAAAAGCTCCTTGAGTGGGTCAACTCAACGGGTGGACTCTACATGACCCACACAGTAGCG   1380
AdTDC19      AAAAAGCTCCTTGAGTGGGTCAACTCAACGGGTGGACTCTACATGACCCACACAGTAGCG   1380
AdTDC20      AAAAAGCTCCTTGAGTGGGTCAACTCAACGGGTGGACTCTACATGACCCACACAGTAGCG   1380
             ******** ***************************************************
```

```
Achn173261   GGTGGACTCTACATGCTGAGGTTTGCGGTGGGGGCTACACTCACAGAAGAGTGTCACGTA   1440
AdTDC1       GGTGGACTCTACATGCTGAGGTTTGCGGTGGGGGCTACACTCACAGAAGAGTGTCACGTA   1440
AdTDC2       GGTGGACTCTACATGCTGAGGTTTGCGGTGGGGGCTACACTCACAGAAGAGTGTCACGTA   1440
AdTDC3       GGTGGACTCTACATGCTGAGGTTTGCGGTGGGGGCTACACTCACAGAAGAGTGTCACGTA   1440
AdTDC4       GGTGGACTCTACATGCTGAGGTTTGCGGTGGGGGCTACACTCACAGAAGAGTGTCACGTA   1440
AdTDC5       GGTGGACTCTACATGCTGAGGTTTGCGGTGGGGGCTACACTCACAGAAGAGTGTCACGTA   1440
AdTDC6       GGTGGACTCTACATGCTGAGGTTTGCGGTGGGGGCTACACTCACAGAAGAGTGTCACGTA   1440
AdTDC7       GGTGGACTCTACATGCTGAGGTTTGCGGTGGGGGCTACACTCACAGAAGAGTGTCACGTA   1440
AdTDC8       GGTGGACTCTACATGCTGAGGTTTGCGGTGGGGGCTACACTCACAGAAGAGTGTCACGTA   1440
AdTDC9       GGTGGACTCTACATGCTGAGGTTTGCGGTGGGGGCTACACTCACAGAAGAGTGTCACGTA   1440
AdTDC10      GGTGGACTCTACATGCTGAGGTTTGCGGTGGGGGCTACACTCACAGAAGAGTGTCACGTA   1440
AdTDC11      GGTGGACTCTACATGCTGAGGTTTGCGGTGGGGGCTACACTCACAGAAGAGTGTCACGTA   1440
AdTDC12      GGTGGACTCTACATGCTGAGGTTTGCGGTGGGGGCTACACTCACAGAAGAGTGTCACGTA   1440
AdTDC13      GGTGGACTCTACATGCTGAGGTTTGCGGTGGGGGCTACACTCACAGAAGAGTGTCACGTA   1440
AdTDC14      GGTGGACTCTACATGCTGAGGTTTGCGGTGGGGGCTACACTCACAGAAGAGTGTCACGTA   1440
AdTDC15      GGTGGACTCTACATGCTGAGGTTTGCGGTGGGGGCTACACTCACAGAAGAGTGTCACGTA   1440
AdTDC16      GGTGGACTCTACATGCTGAGGTTTGCGGTGGGGGCTACACTCACAGAAGAGTGTCACGTA   1440
AdTDC17      GGTGGACTCTACATGCTGAGGTTTGCGGTGGGGGCTACACTCACAGAAGAGTGTCACGTA   1440
AdTDC18      GGTGGACTCTACATGCTGAGGTTTGCGGTGGGGGCTACACTCACAGAAGAGTGTCACGTA   1440
AdTDC19      GGTGGACTCTACATGCTGAGGTTTGCGGTGGGGGCTACACTCACAGAAGAGTGTCACGTA   1440
AdTDC20      GGTGGACTCTACATGCTGAGGTTTGCGGTGGGGGCTACACTCACAGAAGAGTGTCACGTA   1440
             ************************************************************

Achn173261   GTCGCCGCTTGGAAGATAATTAAGGAGGGAGCTGATGTTTTGCTCAGGTCAAAGGATGCT   1500
AdTDC1       GTCGCCGCTTGGAATTTAATTAAGGAGGGAGCTGATGTTTTGCTCAGGTCAAAGGATGCT   1500
AdTDC2       GTCGCCGCTTGGAATTTAATTAAGGAGGGAGCTGATGTTTTGCTCAGGTCAAAGGATGCT   1500
AdTDC3       GTCGCCGCTTGGAATTTAATTAAGGAGGGAGCTGATGTTTTGCTCAGGTCAAAGGATGCT   1500
AdTDC4       GTCGCCGCTTGGAATTTAATTAAGGAGGGAGCTGATGTTTTGCTCAGGTCAAAGGATGCT   1500
AdTDC5       GTCGCCGCTTGGAATTTAATTAAGGAGGGAGCTGATGTTTTGCTCAGGTCAAAGGATGCT   1500
AdTDC6       GTCGCCGCTTGGAATTTAATTAAGGAGGGAGCTGATGTTTTGCTCAGGTCAAAGGATGCT   1500
AdTDC7       GTCGCCGCTTGGAATTTAATTAAGGAGGGAGCTGATGTTTTGCTCAGGTCAAAGGATGCT   1500
AdTDC8       GTCGCCGCTTGGAATTTAATTAAGGAGGGAGCTGATGTTTTGCTCAGGTCAAAGGATGCT   1500
AdTDC9       GTCGCCGCTTGGAAGTTAATTAAGGAGGGTGCTGATGTTTTGCTCAGGTCAAAGGATGCT   1500
AdTDC10      GTCGCCGCTTGGAAGTTAATTAAGGAGGGTGCTGATGTTTTGCTCAGGTCAAAGGATGCT   1500
AdTDC11      GTCGCCGCTTGGAAGTTAATTAAGGAGGGAGCTGATGTTTTGCTCAGGTCAAAGGATGCT   1500
AdTDC12      GTCGCCGCTTGGAAGTTAATTAAGGAGGGAGCTGATGTTTTGCTCAGGTCAAAGGATGCT   1500
AdTDC13      GTCGCCGCTTGGAAGTTAATTAAGGAGGGAGCTGATGTTTTGCTCAGGTCAAAGGATGCT   1500
AdTDC14      GTCGCCGCTTGGAAGTTAATTAAGGAGGGAGCTGATGTTTTGCTCAGGTCAAAGGATGCT   1500
AdTDC15      GTCGCCGCTTGGAAGTTAATTAAGGAGGGAGCTGATGTTTTGCTCAGGTCAAAGGATGCT   1500
AdTDC16      GTCGCCGCTTGGAAGTTAATTAAGGAGGGAGCTGATGTTTTGCTCAGGTCAAAGGATGCT   1500
AdTDC17      GTCGCCGCTTGGAAGTTAATTAAGGAGGGAGCTGATGTTTTGCTCAGGTCAAAGGATGCT   1500
AdTDC18      GTCGCCGCTTGGAAGTTAATTAAGGAGGGAGCTGATGTTTTGCTCAGGTCAAAGGATGCT   1500
AdTDC19      GTCGCCGCTTGGAAGTTAATTAAGGAGGGAGCTGATGTTTTGCTCAGGTCAAAGGATGCT   1500
```

```
AdTDC20       GTCGCCGCTTGGAAGTTAATTAAGGAGGGAGCTGATGTTTTGCTCAGGTCAAAGGATGCT    1500
              **************  ************* ******************************


Achn173261    ACATAG         1506
AdTDC1        ACATAG         1506
AdTDC2        ACATAG         1506
AdTDC3        ACATAG         1506
AdTDC4        ACATAG         1506
AdTDC5        ACATAG         1506
AdTDC6        ACATAG         1506
AdTDC7        ACATAG         1506
AdTDC8        ACATAG         1506
AdTDC9        ACATAG         1506
AdTDC10       ACATAG         1506
AdTDC11       ACATAG         1506
AdTDC12       ACATAG         1506
AdTDC13       ACATAG         1506
AdTDC14       ACATAG         1506
AdTDC15       ACATAG         1506
AdTDC16       ACATAG         1506
AdTDC17       ACATAG         1506
AdTDC18       ACATAG         1506
AdTDC19       ACATAG         1506
AdTDC20       ACATAG         1506
```

Figure S3: Clustal Omega alignment of the candidate *Actinidia deliciosa TDCs*. The number after AdTDC represents the cultivated *Escherichia coli* colony. Achn173261 is the *TDC* sequence of *Actinidia chinensis*.

© 2019 by the authors. Submitted for possible open access publication under the terms and conditions of the Creative Commons Attribution (CC BY) license (http://creativecommons.org/licenses/by/4.0/).
